# Host Innate Antiviral Response to Influenza A Virus Infection: From Viral Sensing to Antagonism and Escape

**DOI:** 10.3390/pathogens13070561

**Published:** 2024-07-03

**Authors:** Wenlong An, Simran Lakhina, Jessica Leong, Kartik Rawat, Matloob Husain

**Affiliations:** Department of Microbiology and Immunology, University of Otago, P.O. Box 56, Dunedin 9054, New Zealand; anwe4100@student.otago.ac.nz (W.A.); laksi167@student.otago.ac.nz (S.L.); leoje827@student.otago.ac.nz (J.L.); rawka371@student.otago.ac.nz (K.R.)

**Keywords:** influenza virus, Toll-like receptors, RIG-I, NOD-like receptors, NLRP3 inflammasome, ZBP1, JAK-STAT, interferons, ISGs, single-nucleotide polymorphism, genetic variants, antiviral therapies

## Abstract

Influenza virus possesses an RNA genome of single-stranded, negative-sensed, and segmented configuration. Influenza virus causes an acute respiratory disease, commonly known as the “flu” in humans. In some individuals, flu can lead to pneumonia and acute respiratory distress syndrome. Influenza A virus (IAV) is the most significant because it causes recurring seasonal epidemics, occasional pandemics, and zoonotic outbreaks in human populations, globally. The host innate immune response to IAV infection plays a critical role in sensing, preventing, and clearing the infection as well as in flu disease pathology. Host cells sense IAV infection through multiple receptors and mechanisms, which culminate in the induction of a concerted innate antiviral response and the creation of an antiviral state, which inhibits and clears the infection from host cells. However, IAV antagonizes and escapes many steps of the innate antiviral response by different mechanisms. Herein, we review those host and viral mechanisms. This review covers most aspects of the host innate immune response, i.e., (1) the sensing of incoming virus particles, (2) the activation of downstream innate antiviral signaling pathways, (3) the expression of interferon-stimulated genes, (4) and viral antagonism and escape.

## 1. Introduction

Host innate immune system is the first line of defense against pathogens, including viruses. It encompasses physical and chemical barriers (e.g., skin and mucous), humoral innate molecules (e.g., lysozymes and cytokines), and cells (e.g., phagocytes) [1]. This system functions in two sequential stages: the sensing (afferent) stage and the effector (efferent) stage. The former is involved in recognizing the infection, while the latter is involved in responding and eliminating the infection [2]. The innate immune system in a host has three tasks: (1) recognize a diverse range of infecting pathogens, (2) respond to infection and kill or eliminate the pathogens, and (3) spare the host tissues while performing tasks 1 and 2 [2]. In addition, the innate immune system also contributes to the activation of the adaptive immune system [1]. Cytokines represent one of the most conserved components of innate immunity and spearhead the host innate immune response against viruses [1,3]. However, against viruses, tasks 2 and 3 of the innate immune response may not be as effective as against other pathogens. Viruses can effectively antagonize or evade the host innate immune response [4,5]. Furthermore, a hyperactive innate immune response can damage the host tissues and harm the host [6,7]. In this review, we have summarized tasks 1 and 2 of the cytokine-mediated host innate immune response to influenza virus infection and how the influenza virus antagonizes and escapes that response.

The influenza virus is the prototypic member of the family *Orthomyxoviridae* and is an enveloped virus with a segmented, linear, negative-sensed, single-stranded RNA genome [8]. Influenza virus exists in four types: A, B, C, and D. Influenza A virus (IAV) has a broad host range, infecting humans, other mammals, and various avian species. Influenza B virus (IBV) and influenza C virus (ICV) primarily infect humans. Influenza D virus (IDV) is the recently isolated influenza virus and is known to infect pigs and cattle [9]. IAV and IBV cause recurring seasonal epidemics in the human population [10,11]. IAV also causes occasional pandemics and sporadic zoonotic outbreaks in the human population [11,12]. The IAV undergoes regular inter-species transmission, particularly between humans, pigs, and avian species, and is the most diverse among influenza viruses [13]. Consequently, due to its relevance to human health, IAV is the most studied influenza virus and is the focus of this review.

The IAV genome is packaged as eight viral ribonucleoprotein (vRNP) complexes [14]. Each vRNP contains one of the eight RNA genome segments, nucleoprotein (NP), and three RNA-dependent RNA polymerase subunits: polymerase acidic (PA), polymerase basic 1 (PB1), and 2 (PB2). The eight vRNPs are surrounded by a host-derived lipid membrane (envelope), which is supported by an underlying layer of matrix 1 (M1) protein. The envelope is decorated with the receptor-binding protein, hemagglutinin (HA), a sialidase, neuraminidase (NA), and an ion-channel, matrix 2 (M2) protein [14]. To infect a host cell, IAV binds to the sialic acid receptor through HA and enters the cell via endocytosis. The viral envelope then fuses with the endosomal membrane, and the vRNPs are released inside the cytoplasm. The vRNPs are then trafficked to the nucleus, where viral RNA transcription and replication take place [14]. The viral mRNAs are exported to the cytoplasm where they are translated into up to 17 viral proteins—eight structural proteins (HA, M1, M2, NA, NP, PA, PB1, and PB2) and nine non-structural proteins (M42, NS1, NS2 or NEP, NS3, PA-X, PA-N155, PA-N182, PB1-F2, and PB1-N40) [15]. The NP, PA, PB1, and PB2 are imported back to the nucleus to assemble vRNPs. The M1 and NEP are also imported into the nucleus to facilitate the nuclear export of vRNPs, which are then trafficked to the plasma membrane [14]. The HA, NA, and M2 are directly transported to the plasma membrane via ER-Golgi transport [14]. At the plasma membrane, all viral components assemble into viral progeny, which are released from the cell by budding [14].

## 2. Sensing of IAV Infection by Host Cells

**Figure 1:**
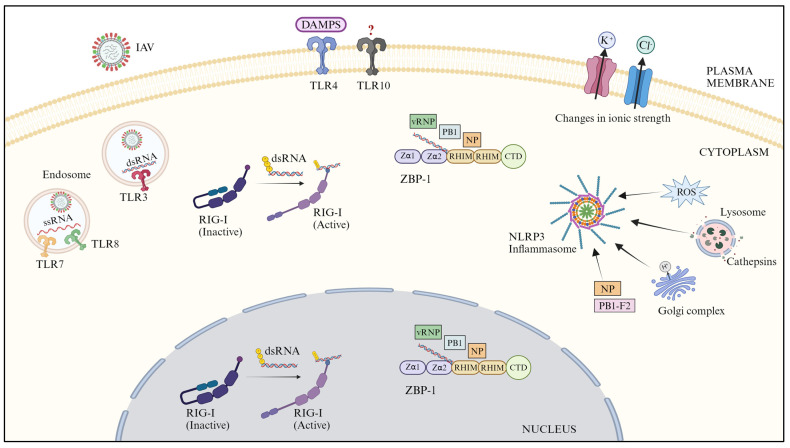
Sensing of IAV infection by PRRs—TLRs, RIG-I, ZBP-1, and NLRP3 inflammasome (created with BioRender.com). Arrows pointing to NLRP3 inflammasome indicate the DAMPs sensed by this PRR; question mark (?), indicate the unknown ligand.

Host innate immune response is the first line of defense against virus infection, and the first stage of this is the sensing or detection of virus infection. Host cells sense virus infection via the pattern recognition receptors (PRRs). PRRs sense different stages of the virus infection in different intracellular compartments of different cell types. Multiple classes of PRRs are known, namely, Toll-like receptors (TLRs), retinoic acid-inducible gene-like receptors (RLRs), nucleotide-binding oligomerization domain (NOD)-like receptors (NLRs), cyclic GMP-AMP synthase (cGAS), and Z-DNA-binding protein 1 (ZBP1). Human cells are known to employ TLRs, RLRs, NLRs, and ZBP1 to sense the IAV infection (Figure 1).

### 2.1. Toll-like Receptors (TLRs)

TLRs are germline-coded, membrane-bound proteins and expressed by both immune and non-immune cells. TLR genes were initially characterized in *Drosophila* embryo development [16,17] and its anti-microbial response [18]. Soon after, human homologs of *Drosophila* TLRs were identified and characterized for how they induce the innate immune response [19,20]. Subsequently, bacterial lipopolysaccharide (LPS) was discovered as the stimulant of TLRs [21,22,23,24,25]. Now, TLRs are known to sense various microbes, including viruses, through their specific molecular signatures, called pathogen-associated molecular patterns (PAMPs) [26].

TLRs are type I transmembrane proteins with an extracellular leucine-rich repeat (LRRs) for the detection of the PAMPs, a transmembrane domain for membrane insertion, and a cytoplasmic Toll/IL-1 receptor (TIR) domain for the downstream signaling [27]. Humans encode 10 TLRs (TLRs 1–10), which, based on their intracellular localization, are categorized into two groups. Group 1 comprises TLR1, TLR2, TLR4, TLR5, TLR6, and TLR10, which localize to the plasma membrane, and group 2 contains TLR3, TLR7, TLR8, and TLR9, which localize to the endosomes. The plasma membrane-localized TLRs recognize membrane-associated PAMPs, whereas the endosomal TLRs detect microbial nucleic acids, including of viruses [26,28].

IAV enters the human cells by endocytosis. Hence, IAV is sensed by TLR3 [29,30] through its double-stranded RNA, TLR7 [31,32,33,34], and TLR8 [33] through its single-stranded RNA (Figure 1). However, TLR4 also senses the IAV infection through damage-associated molecular patterns (DAMPs), which are released from IAV-infected cells [35,36]. Furthermore, TLR10 may also sense the IAV through a yet-to-be-known ligand [37] (Figure 1). 

### 2.2. Retinoic Acid-Inducible Gene-like Receptors (RLRs)

RLRs are RNA helicases, which predominantly localize to the cytoplasm of most cell types. Three RLRs—retinoic acid-inducible gene-1 (RIG-I), melanoma differentiation-associated gene 5 (MDA5), and laboratory of genetics and physiology 2 (LGP2), are known. All RLRs are characterized by the presence of a central helicase domain and a C-terminal domain (CTD) for detecting the PAMPs. In addition, RIG-I and MDA5 possess two amino-terminal caspase activation and recruitment domains (CARDs) for downstream signaling [38]. RIG-I is the prototypic RLR and was first identified as one of the retinoic acid-inducible genes in differentiated leukemia cells [39,40]. Later, LPS was also found to induce the RIG-I expression [41]. Furthermore, a porcine homolog of RIG-I was found to be induced by the infection of porcine reproductive and respiratory syndrome virus (PRRSV) [42]. In 2004, Yoneyama et al. demonstrated that RIG-I senses double-stranded RNA (dsRNA) and induces the innate immune response against RNA viruses [43]. Subsequently, RIG-I was found to detect the dsRNA of multiple RNA viruses, including that of IAV [44,45]. Specifically, RIG-I senses short partially dsRNA strands containing base-paired, blunt-ended 5’ ends with a tri- or di-phosphate group, also known as a panhandle structure [46,47,48,49,50,51,52]. Such partially dsRNA panhandle structures in the life cycle of single-stranded RNA (ssRNA) viruses, like IAV, are generated during viral genome transcription and replication [53]. The MDA5 also senses similar but longer and virus-specific dsRNA architecture [45,51,54].

RIG-I has been shown to sense multiple forms of IAV replicating and transcribing RNAs through their panhandle-type architecture [47,52,55,56,57] (Figure 1). However, unlike most RNA viruses, IAV replicates its RNA genome in the host cell nucleus. Evidently, RIG-I is also localized to the nucleus [58] (Figure 1). There remains a scarcity of reports identifying MDA5 and/or LGP2 as the direct sensor of IAV RNA.

### 2.3. Z-DNA-Binding Protein 1 (ZBP1)

ZBP1 (also known as DAI) is the latest in PRRs and was identified as DLM-1 in tumor-activated macrophages [59]. DLM-1 was renamed as ZBP1 after the discovery that it possesses a Z-DNA-binding domain [60] and binds Z-DNA—a left-handed, double-stranded DNA helix [61,62]. A PRR function of ZBP1 was first reported when it was discovered to sense DNA as a PAMP and induce an innate immune response [63,64]. Later, ZBP-1 was also found to sense the RNA [65]. ZBP1 contains two N-terminal Z-nucleic acid binding domains (Zα1 and Zα2), a Z-DNA-binding domain (D3) next to Zα2, two central receptor-interacting protein homotypic interaction motif (RHIM) domains, and a C-terminal signal domain (SD) [60,66,67]. 

Like RIG-I, ZBP1 is mainly a cytosolic protein but is also localized to the nucleus [62,68,69,70]. ZBP1 senses the IAV infection by sensing multiple viral PAMPs—RNA, NP, and PB1 proteins as well as vRNP complex, both in the cytoplasm and in the nucleus [69,70,71,72,73] (Figure 1).

### 2.4. NOD-like Receptors (NLRs)

NLRs are also the recently described intracellular sensors of microbial PAMPs and DAMPs. The class II transactivator (CIITA) was the first NLR to be identified [74]. Now, 22 NLRs are known [75], and, phylogenetically, they are divided into three subfamilies: NOD, NLRP, and IPAF [76]. NLRs are characterized by the presence of N-terminal caspase recruitment (CARD) or pyrin (PYD) domain, a central nucleotide-binding and oligomerization domain (NACHT), and C-terminal leucine-rich repeats (LRRs) [76]. Upon sensing various PAMPs and DAMPs, NLRs oligomerize and form a multiprotein scaffold or platform, called the inflammasome [76]. The NLR family pyrin domain-containing 3 (NLRP3) inflammasome is the most characterized and significant among inflammasomes because it senses a diverse range of PAMPs and DAMPs [77].

Unlike TLRs and RIG-I, the NLRP3 inflammasome seems to sense the IAV infection through DAMPs rather than PAMPs. These DAMPs include (1) changes in the intracellular reactive oxygen species production [78] and ionic concentration [79], and lysosome function [78], (2) integrity or order of the Golgi complex [80] and viral proteins, such as PB1-F2 [81,82] and NP [83] (Figure 1). The sensing of NP is also facilitated by the host restriction factor MxA [83].

## 3. Downstream Innate Immune Signaling against IAV

### 3.1. TLR-Mediated Downstream Signaling

After detecting the PAMPs, TLRs signal via either myeloid differentiation primary response 88 (MyD88)-dependent pathway or Toll–interleukin 1 (IL-1) receptor (TIR)-domain-containing adaptor inducing interferon (IFN)-β (TRIF)-dependent pathway.

In the MyD88 pathway, TLRs dimerize and recruit MyD88 via TIR domains. Then, MyD88 interacts with IL-1R-associated kinase 4 (IRAK4) through death domains (DDs) and activates the IRAK1 [84], forming a complex called the “Myddosome” [85]. Subsequently, IRAK1 activates the ubiquitin ligase, tumor necrosis factor (TNF) receptor-associated factor 6 (TRAF6) [86,87], which then ubiquitinates and activates the TGFβ-activated kinase 1 (TAK1) [87,88]. Activated TAK1 phosphorylates and activates the mitogen-activated protein kinases (MAPKs) and inhibitor of NF-κB (IκB) kinase (IKK) complex, which comprises IKKα, IKKβ, and NF-κB essential modulator (NEMO) [86,88,89]. The activated MAPKs then phosphorylate the transcription factor activator protein-1 (AP-1). On the other hand, the IKK complex phosphorylates interferon-regulatory factor (IRF) 5 and 7 as well as IκB. The phosphorylation of IκB leads to its ubiquitination and, subsequently, its proteasome-mediated degradation. This causes the release of NF-κB from IKK complex [90,91,92,93,94]. The phosphorylated AP-1, IRFs 5 and 7, and released NF-κB then translocate to the nucleus.

In the TRIF pathway, TLRs also dimerize and recruit TRIF, which then activates TRAF3 and TRAF6. Subsequently, TRAF3 interacts with TRAF family member-associated NF-κB activator (TANK)-binding kinase 1 (TBK1) and inhibitor of κB (IκB) kinase-related kinase-ε (IKKε), which, in turn, phosphorylates the transcription factor IRF3. On the other hand, TRAF6 ubiquitinates receptor-interacting serine/threonine-protein kinase 1 (RIPK-1), which then phosphorylates TAK1. The result of these pathways is also the phosphorylation of AP-1 or IRFs, or release of NF-κB, and their subsequent translocation to the nucleus [95,96,97,98,99,100,101,102,103,104]. In the nucleus, AP-1, the IRFs, and NF-κB engage with their respective promoters and stimulate the expression of cytokines, interferons (IFNs), and pro-inflammatory cytokines.

**Figure 2:**
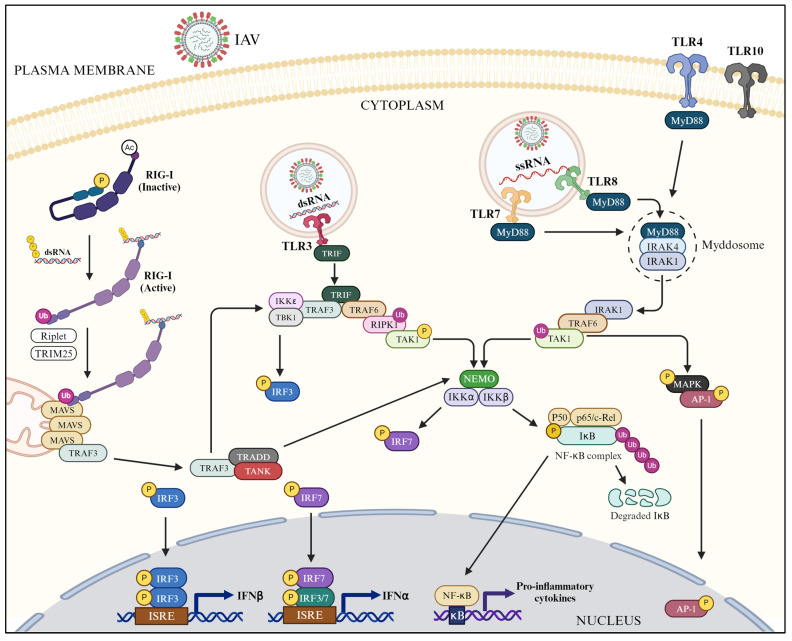
The TLR-mediated and RIG-I-mediated downstream signaling in IAV-infected cells (created with BioRender.com).

As stated above, IAV infection is primarily sensed by the TLRs 3, 7, and 8. Both TLR 7 and 8 signal via the MyD88-dependent pathway, whereas TLR3 signals via TRIF-dependent pathway [26,28]. The canonical TLR3 [29,34,105,106,107,108,109] and TLR7 [31,32,33,105,110] signaling has been reported to occur during IAV infection (Figure 2). Furthermore, TLR4, which senses DAMPs from IAV-infected cells, also signals through the MyD88-dependent pathway [35] (Figure 2).

### 3.2. RLR-Mediated Downstream Signaling

RIG-I is present in an inactivated form in uninfected cells and in the absence of an RNA agonist. In this form, RIG-I is phosphorylated with sequestered CARDs [111,112,113,114,115,116]. Upon binding the viral RNA via CTD and helicase domain, RIG-I undergoes dephosphorylation and ATP-dependent conformational changes, which release the CARDs [116,117,118,119,120,121,122]. The CARDs then undergo sequential ubiquitination by multiple ubiquitin ligases, such as TRIM25 [123,124,125,126,127]. Such ubiquitination leads to the oligomerization and activation of RIG-I [128,129,130]. Activated RIG-I is then translocated to mitochondrial, peroxisomal, or mitochondrial-associated membranes, where it binds to mitochondrial antiviral signaling protein (MAVS) [131,132,133,134]. This interaction leads to the oligomerization and activation of MAVS [135]. Activated MAVS then forms a complex with TRAF3 (which ubiquitinates RIPK1), TNFR-associated death domain (TRADD) protein, and TANK to facilitate the activation and nuclear translocation of NF-κB and IRFs 3 and 7, which leads to the expression of IFNs α, β, or γ as well as pro-inflammatory cytokines [136,137,138]. The MDA5 gets activated and signals in a manner similar to RIG-I [111,121,130]. In contrast, LGP2, which binds dsRNA [139,140] but lacks CARDS, may sequester the dsRNA to antagonize RIG-I signaling [140,141,142,143]. However, LGP2 acts as a co-factor of MDA5 and promotes MDA5 signaling [54,139,143,144,145,146,147,148].

After sensing IAV infection, RIG-I signals via the canonical pathway described above, leading to the expression of IFNs and pro-inflammatory cytokines [34,45,47,101,106,129,149,150,151,152,153] (Figure 2). In addition to humans and mice, such anti-IAV RIG-I signaling has also been observed in ducks, waterfowl, and dogs [154,155,156,157,158] but not in chickens and turkeys, which lack RIG-I and some ubiquitin ligases [154,155,159]. Furthermore, NP of avian IAV H7N9 subtype can activate RIG-I signaling by binding directly to and stabilizing TRAF3 [160]. In addition, various co-factors, both proteins and non-coding RNAs (ncRNAs), such as E3 ligase FBXW7, deubiquitylase OTUB1, histone deacetylase 6 (HDAC6), RNA helicases DDX6 and DHX16, miRNA-136, lncRNA HCG4, and U1 snRNA, have been identified to augment RIG-I signaling in IAV-infected cells [161,162,163,164,165,166,167,168,169,170,171,172,173]. Interestingly, the infection-induced re-arrangement of the actin cytoskeleton can also activate the RIG-I and downstream signaling through the activation of protein phosphatase-1 and dephosphorylation of RIG-I [169].

MDA5 also signals through the RIG-I-like canonical pathway and induces the production of IFNs and pro-inflammatory cytokines in response to IAV infection in different host species [111,174,175,176]. In chickens that lack RIG-I, MDA5 is the main RLR to sense the IAV infection and induce the innate antiviral response [177,178]. In contrast, LGP2 seems to promote IAV infection by downregulating the innate antiviral response in infected cells [179,180].

### 3.3. ZBP1-Mediated Downstream Signaling

ZBP1 is an interferon-stimulated gene [63] and, upon binding the PAMPs or DAMPs, signals via multiple pathways, which leads to type I IFN expression and programmed cell death or PANoptosis [67]. In one pathway, ZBP1 is activated through phosphorylation by TANK-binding kinase 1 (TBK1) [181]. The activated ZBP1-TBK1 scaffold then recruits and phosphorylates IRF3, which then translocates to the nucleus and initiates type I IFN expression [63]. In another pathway, ZBP1 signals through the RIPK1–RIPK3 axis and activates NF-kB [64]. Additionally, ZBP1 signaling via RIPK1–RIPK3 axis also involves the caspase 8, which leads to PANoptosis—the caspase 8-mediated apoptosis, the NLRP3 inflammasome-mediated pyroptosis, and the RIPK3-MLKL-mediated necroptosis of cells [65,67,182].

**Figure 3:**
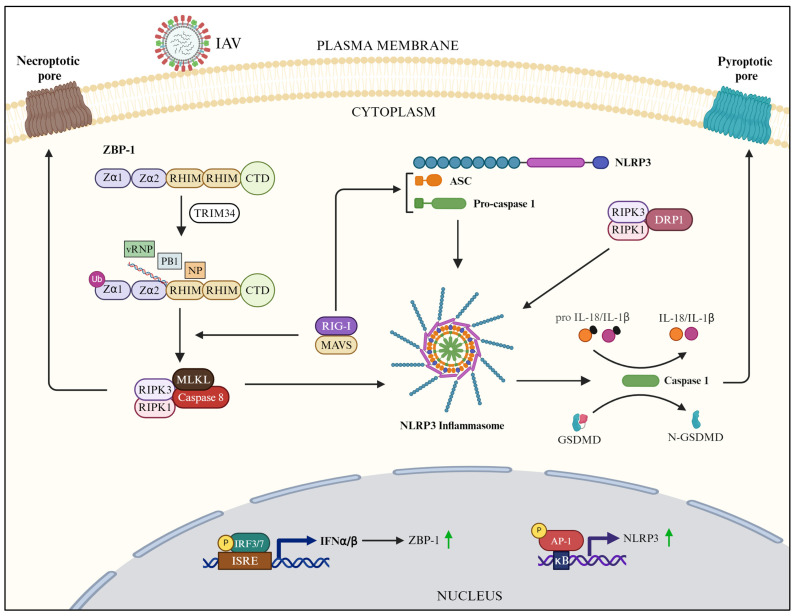
ZBP-1-mediated and NLRP3 inflammasome-mediated downstream signaling in IAV-infected cells (created with BioRender.com).

During IAV infection, ZBP1 expression is upregulated in an IFN-dependent manner [69,183] (Figure 3). Furthermore, ZBP1 is ubiquitinated by TRIM34 and activated by the RIG-I-MAVS pathway [72,184] (Figure 3). Activated ZBP1 then senses various IAV PAMPs and DAMPs (as described above) and signals via the RIPK1–RIPK3–MLKL–caspase 8 axis and induces the PANoptosis of infected cells [69,70,71,185] (Figure 3). Caspase 6 is also an important cofactor in this axis [186]; however, it has been reported that this axis can also function independently of MLKL [187,188].

### 3.4. NLRP3-Mediated Downstream Signaling

NLRP3-mediated signaling comprises five main components: NLRP3, apoptosis-associated speck-like protein containing a caspase-recruitment domain (ASC), interleukin (IL)-1β, IL-18, and caspase-1. The IL-1β, IL-18, and caspase-1 exist in an inactive “pro” form in unstimulated cells. NLRP3-mediated signaling is a two-step process. The first step is priming, where expression of NLRP3, pro-IL-1β, and pro-IL-18 is induced by pathways activated by other PRRs [189,190]. The second step is the formation and activation of the inflammasome, where, after sensing various DAMPs, NLRP3 oligomerizes and forms a complex with ASC. Then, pro-caspase-1 is recruited to this complex and becomes activated through self-cleavage. Subsequently, active caspase-1 cleaves pro-IL-1β and pro-IL-18 into active forms, which activate the pro-inflammatory and adaptive immune responses [191]. In addition, caspase-1 cleaves gasdermin D, which then triggers the pyroptosis of cells [189].

In IAV-infected cells, multiple mechanisms of NLRP3 inflammasome priming and activation have been described, with the involvement of several cofactors. It has been shown that IFN-inducible GTPase, IRGB10 [192], and transcription factor AP-1 [190] prime the NLRP3 inflammasome in IAV-infected cells (Figure 3). Furthermore, RIG-I activates NLRP3 inflammasome in IAV-infected cells by interacting with caspase-1 and ASC [193] (Figure 3). However, NLRP3 inflammasome can also be activated independent of RIG-I through the RIPK1–RIPK3–dynamin-related protein 1 (DRP1) axis [194,195] (Figure 3). Furthermore, ZBP1 can activate NLRP3 inflammasome in IAV-infected cells through the RIPK1–RIPK3–caspase-8 axis (Figure 3), where MLKL can be dispensable [69,187,188]. Nevertheless, both MLKL and caspase-8 can independently activate NLRP3 inflammasome in IAV-infected cells [188]. Therefore, it is plausible to say that, in IAV-infected cells, NLRP3 inflammasome is activated by the ZBP1–RIPK1–RIPK3–DRP1/caspase-8 axis. Furthermore, DEAD-box helicase 3 X-linked (DDX3X) [196], TRIM25 [197], and mitochondrial protein mitofusin 2 [198] augment the activation of NLRP3 inflammasome in IAV-infected cells.

### 3.5. IRFs in Downstream Signaling

IRFs are a nine-member family (IRFs 1–9) of transcription factors. All IRFs possess a conserved N-terminal DNA-binding domain with tryptophan repeats that bind to IFN-stimulated response elements (ISREs). The IRFs 3, 5, and 7 are most studied in the context of innate antiviral signaling and are involved in both RLR-mediated and TLR-mediated (MyD88-dependent and TRIF-dependent) downstream signaling [199]. After phosphorylation, IRFs dimerize and translocate to the nucleus, where they form a complex with histone acetyltransferases, such as p300/CBP [199,200,201,202,203]. The complex then binds to the ISREs in the promoter region of type I IFN genes and recruits an “enhanceosome” in order to initiate the transcription [204].

IRFs, 3, 5, and 7 have all been implicated in innate antiviral response during IAV infection [205,206,207,208,209,210] (Figure 2). Particularly, IRF3 and IRF7 seem to work in tandem [205,211] (Figure 2) and utilize annexin-A1 and cellular nucleic acid-binding protein (CNBP) as co-factors [212,213] for efficient activation and signaling to initiate the production of type I IFNs in IAV-infected cells. In addition, IRF1 has been shown to contribute to innate antiviral response during IAV infection [207,214,215]; one of the mechanisms for this is the positive regulation of ZBP1 expression and, consequently, ZBP1-mediated activation of NLRP3 inflammasome [215]. 

### 3.6. NF-κB in Downstream Signaling

NF-κB is a family of five (p50, p52, p65 or RelA, c-Rel, and RelB) transcription factors. All five possess a conserved N-terminal Rel homology region (RHR), which enables their dimerization and binding to DNA. Furthermore, RelA (p65), RelB, and c-Rel possess a C-terminal transactivation domain (TAD), which activates the transcription of their target genes [90]. After release from IKK complex, the NF-κB proteins dimerize and translocate to the nucleus. Here, NF-κB proteins bind to 9–11 base pair DNA nucleotide sequences, called κB sites, present in the promoter/enhancer region of various genes, including proinflammatory cytokines and chemokines, and initiate their transcription [216,217,218] (Figure 2).

NF-κB has been described to play a critical role in IAV-induced host innate immune response [219,220]. Furthermore, cofactors like FK506-binding protein 5 (FKBP5) [151] and Jade family PHD zinc finger 3 (JADE3) [221] enhance NF-κB signaling by enhancing either the release [222] or the nuclear translocation of NF-κB [221] in IAV-infected cells. 

### 3.7. Interferons (IFNs)

IFNs are the main cytokines that combat viral infections. Three types of IFNs, I, II, and III, are known. Type I consists of IFN-α (multiple subtypes), IFN-β, IFN-ε, IFN-κ, and IFN-ω, type II consists of IFNγ, whereas type III consists of IFN-λ1 (IL-29), IFN-λ2 (IL-28A), IFN-λ3 (IL-28B), and IFN-λ4 [4]. Type I and III IFNs are expressed and secreted from infected cells via TLR- and RLR-mediated downstream signaling. Secreted IFNs then bind to infected and uninfected cells in an autocrine and paracrine manner, respectively, through their respective receptors, e.g., IFN alpha receptor (IFNAR) for type I IFNs and IFN lambda receptor (IFNLR) for type III IFNs [4]. This activates the Janus kinase (JAK)–signal transducer and activator of the transcription (STAT) pathway, resulting in the expression of hundreds of IFN-stimulated genes (ISGs), which create an “antiviral state” in IAV-infected cells [223,224]. Both type I and type III IFNs are produced by IAV-infected cells [211,225]. However, type III IFNs are the predominant IFNs in IAV-infected cells and are produced first, followed by the type I IFNs [226,227,228].

### 3.8. JAK-STAT Pathway

Cytokine receptors, JAKs, and STATs are three main components of the JAK-STAT pathway. The cytokine receptors are plasma membrane-localized membrane proteins with an extracellular domain, a transmembrane domain, and an intracellular domain. The JAKs are cytoplasm-localized protein kinases and exist in four types—JAK1, JAK2, JAK3, and TYK2. JAKs contain an N-terminal four-point-one, ezrin, radixin, and moesin (FERM) domain followed by the Src Homology 2 (SH2) and pseudokinase domains, and a C-terminal kinase domain. The STATs are transcription factors and localize to the cytoplasm in their inactive form. STATs exist in seven types—STAT1, STAT2, STAT3, STAT4, STAT5a, STAT5b, and STAT6—and contain several domains: an N-terminal domain (ND) followed by a coiled-coil domain (CCD), a DNA-binding domain (DBD), a linker domain (LK), a Src Homology 2 domain (SH2), and a C-terminal transactivation domain (AD). The cytokine receptors exist as dimers and remain associated with two molecules of JAKs through the interaction between their intracellular domain and JAKs’ FERM domain [229,230,231]. 

When cytokines, like IFNs, engage with their receptors, the two molecules of JAKs are auto activated by trans-phosphorylation. Activated JAKs then phosphorylate the intracellular domains of cytokine receptors, which creates the docking sites for STATs in JAK-cytokine receptor complex. Docked STATs are then phosphorylated by JAKs and dissociate themselves from the complex. The dissociated phosphorylated STATs then form a homodimer or heterodimer [230], which, in turn, binds IRF9 and forms the IFN-stimulated gene factor 3 (ISGF3) complex [232]. The ISGF3 then translocate to the nucleus, where it binds to ISREs of ISGs and initiates their transcription [229,230].

**Figure 4:**
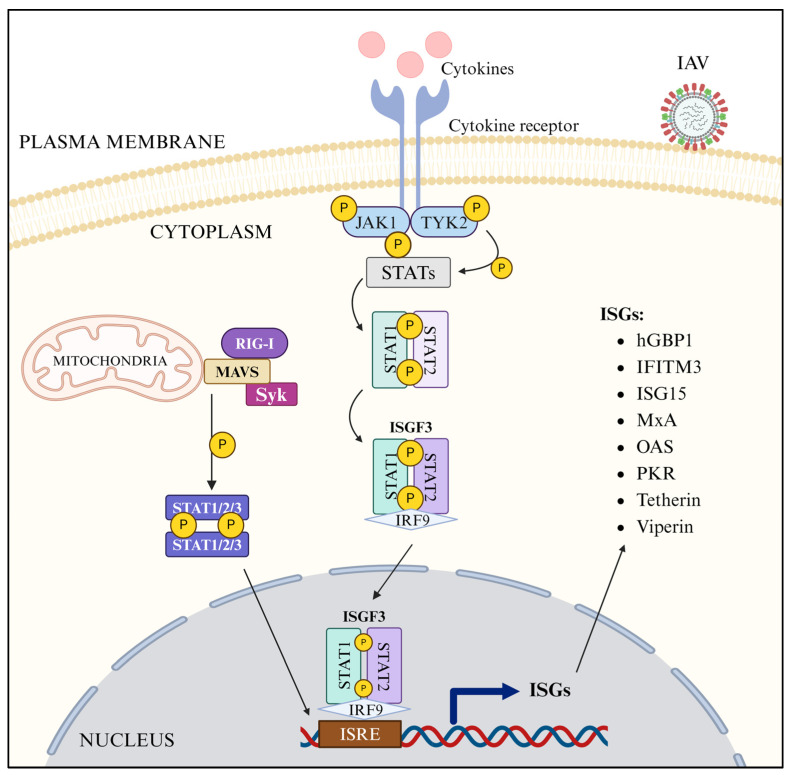
JAK-STAT signaling in IAV-infected cells (created with BioRender.com).

Above canonical JAK-STAT pathway, with the individual involvement of STAT1, STAT2, or STAT3 [233,234,235,236], has been described to be activated in IAV-infected cells, producing many ISGs [224] (Figure 4). However, the STAT1-mediated, STAT2-mediated, or STAT3-mediated antiviral response can also be activated in IAV-infected cells independent of cytokine receptor signaling via the RIG-I-MAVS axis [234,235,236] (Figure 4).

### 3.9. ISGs

Many ISGs, including proteins and ncRNAs, have been identified to express in IAV-infected cells (Figure 4) and described to inhibit IAV infection at different stages of its life cycle [224]. The ISGs, MUC1, and SPOCK2 inhibit the attachment of IAV virus particles to the host cell surface. The ISGs, hGBP-2, hGBP-5, IFITM 1, 2, and 3, NCOA7, RABGAP1L, and Serpin 1 inhibit IAV entry to host cells. The ISGs, CEACAM1, miRNA101, hGBP-3, p21, SERTAD3, IFIT 1, 2, and 3, ISG20, OAS 1, 2, 3, and L, TRIM 25 and 56, MOV10, MxA, PKR, and ZAPS inhibit the synthesis of either viral mRNAs or viral proteins, whereas the ISGs, ISG15, TRIM 14, 21, 22, and 35, and ZAPL target various host and viral proteins and inhibit IAV assembly. The ISGs, tetherin, and viperin inhibit IAV release. Finally, the ISGs, IFI16, miRNA485, circVAMP3, ISG15, lncRNAs, and SLFN 11 and 14 promote different stages of the host innate antiviral response against IAV [224]. 

### 3.10. Pro-Inflammatory Cytokines, Chemokines, and Growth Factors

Pro-inflammatory cytokines such as IL-1 and IL-18, chemokines such as IL-6, and growth factors such as G-CSF are produced through NF-κB signaling [218]. These molecules protect the lungs from IAV-induced inflammation, promote IAV clearance from the lungs, and increase host survival [237,238,239,240,241]. Furthermore, IL-1 and IL-18 promote a host-adaptive immune response against IAV [191,241,242,243]. However, the hyperactivation of these molecules during IAV infection, particularly under pre-existing medical conditions [244] or infection with an avian IAV [245], can increase lung injury and disease severity [242,246].

## 4. Antagonism of Host Innate Immune Response by IAV

**Figure 5:**
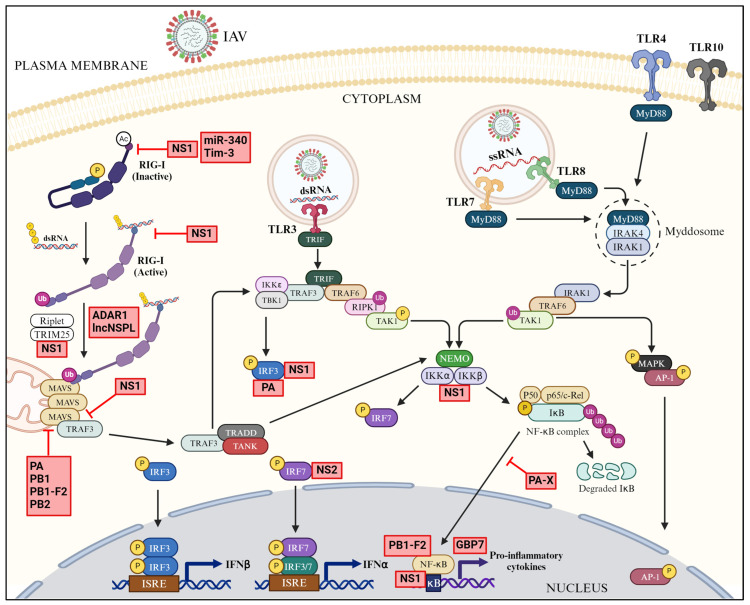
Antagonism of RIG-I-mediated and TLR-mediated downstream signaling by IAV (created with BioRender.com).

**Figure 6:**
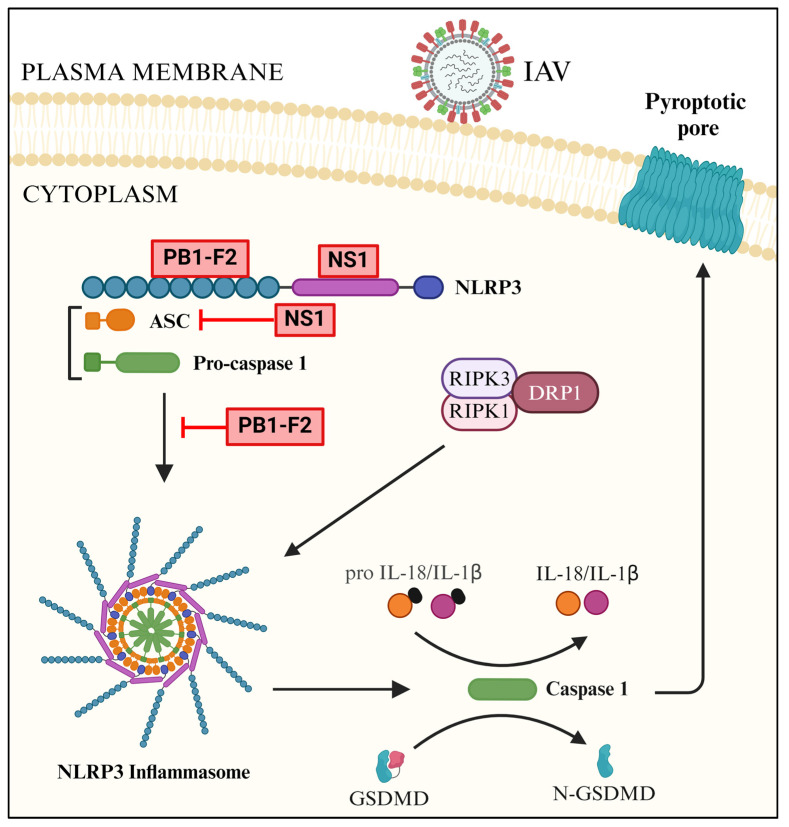
Antagonism of NLRP3 inflammasome-mediated downstream signaling by IAV (created with BioRender.com).

**Figure 7:**
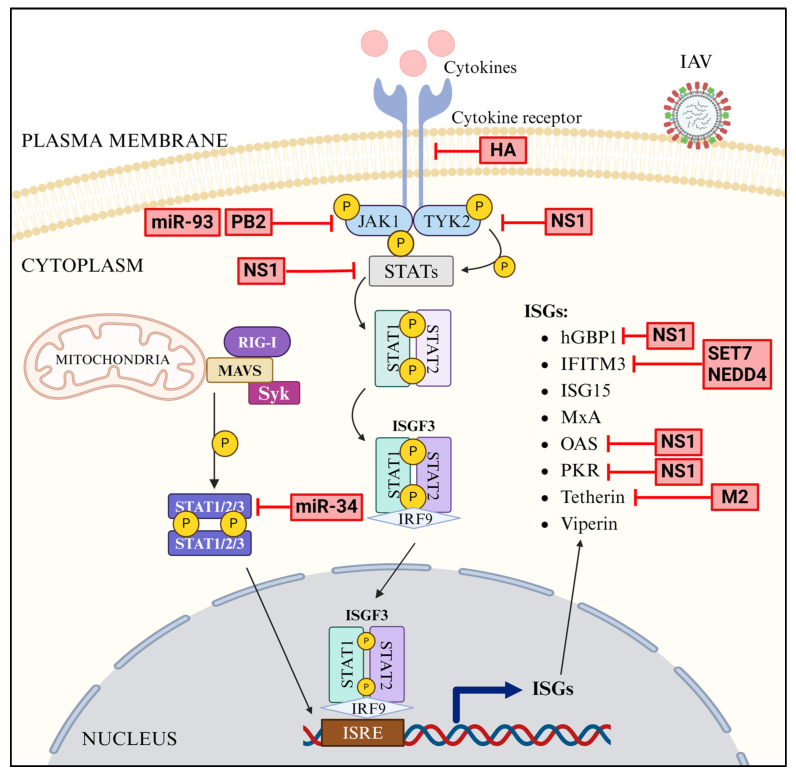
Antagonism of JAK-STAT signaling by IAV (created with BioRender.com).

Even though the host employs a multipronged innate antiviral response to restrict or eliminate the virus infection, viruses have evolved their own effective strategies to antagonize such response and multiply. These strategies include sequestration, degradation, downregulation of the expression, and interference in the function of the components of innate immune response. As described above, the host employs multiple pathways to exert a concerted antiviral response to restrict or eliminate IAV infection. In turn, IAV has evolved multiple mechanisms through the deployment of either viral or host proteins or host ncRNAs to effectively antagonize those host innate antiviral pathways (Figure 5, Figure 6 and Figure 7).

### 4.1. Antagonism of RIG-I

IAV antagonizes RIG-I-mediated sensing and signaling by multiple mechanisms and by employing both viral and host proteins (Figure 5).

IAV primarily employs non-structural 1 (NS1) protein, its main virulence factor, to antagonize the RIG-I-mediated signaling at multiple stages and by multiple mechanisms (Figure 5). NS1 is a multi-functional protein, possessing an N-terminal RNA-binding domain (RBD) followed by a linker region (LR), an effector domain (ED), and a C-terminal tail (CTT) [247]. Firstly, NS1 downregulates the expression of RIG-I in infected cells (Figure 5), either directly or indirectly, by (1) binding to RIG-I pre-mRNA and interfering with its maturation [248], (2) promoting the recruitment of the transcriptional repressor, CCAAT/enhancer binding protein beta (C/EBPβ) to RIG-I promoter [249], and (3) upregulating the expression of the RNase, monocyte chemotactic protein-induced protein 1 (MCPIP1) [250]. Secondly, NS1 interacts with RIG-I and competes for viral RNA binding, consequently blocking the viral RNA sensing [47,149,251,252,253,254] (Figure 5). Furthermore, NS1 interacts with and sequesters the ubiquitin ligases, TRIM25 and Riplet (Figure 5), blocking the RIG-I ubiquitination and, consequently, its activation [255,256,257,258]. NS1 also blocks RIG-I-mediated signaling further downstream. NS1 sequesters the RIG-I co-factors 14-3-3ε, OTUB1, and signaling molecule TRAF3 and interferes with the translocation of RIG-I to mitochondrial membrane [164,259] and the formation of MAVS–TRAF3 complex [260,261] (Figure 5), respectively.

PB1-F2 is another non-structural protein that IAV employs to antagonize the RIG-I signaling. PB1-F2 is a small protein encoded by +1 open reading frame of the IAV PB1 gene segment and localizes to the mitochondria [262,263]. Through localization to mitochondria, PB1-F2 binds MAVS and impairs downstream RIG-I signaling [262,263,264,265,266,267] (Figure 5). In addition, PB1-F2 causes the mitochondrial fragmentation and mitophagy [263,268].

IAV employs auxiliary functions of three RNA polymerase subunits—PA, PB1, and PB2—to antagonize the RIG-I signaling. For this, PA, PB1, and PB2 target MAVS (Figure 5) and either sequester it or promote its degradation [269,270,271]. Further, PA and PB1 can directly bind to and antagonize the RIG-I [272]. 

In addition, IAV recruits host proteins and ncRNAs such as K-homology-splicing regulatory protein (KHSRP) [273], an adenosine deaminase acting on RNA (ADAR1) isoform [274] and lncNSPL [275] to prevent the RIG-I activation, and miR340 [276] and T-cell immunoglobulin and mucin protein-3 (Tim-3) [277] to downregulate the RIG-I expression in infected cells (Figure 5). Furthermore, IAV degrades host HDAC6 [278] and progesterone receptor membrane component-1 (PGRMC1) [279], which promote RIG-I activation [173,279].

### 4.2. Antagonism of IRFs and NF-κB

Multiple IAV proteins antagonize IRF3 and IRF7. NS1 interacts with both IRF3 and IRF7 and inhibits their activation [280,281]. The NS2 protein (now known as nuclear export protein or NEP) of IAV also interacts with IRF7 and blocks its nuclear translocation [282] (Figure 5). Furthermore, viral RNA polymerase subunit, PA interacts with IRF3 and inhibits its activation [283] (Figure 5).

NS1 antagonizes NF-κB by interacting with IKK complex and blocking the degradation of IκB and release of NF-κB [284,285] (Figure 5). Furthermore, NS1 competes with NF-κB for binding to IFN λ promoter [286] (Figure 5). Also, PB1-F2 interferes with the binding of NF-κB to DNA [220] (Figure 5). PA-X, another IAV non-structural protein encoded by the PA gene segment through ribosomal frameshifting [287], antagonizes NF-κB by blocking its nuclear translocation [288] (Figure 5). In addition, IAV recruits the guanylate-binding protein 7 (GBP7) to inhibit NF-κB signaling in infected cells [289] (Figure 5).

### 4.3. Antagonism of NLRP3

IAV employs NS1 to antagonize the NLRP3-mediated signaling. NS1 interacts with NLRP3 and prevents NLRP3 inflammasome activation and, consequently, caspase 1 and IL-1β activation [290,291,292,293]. Furthermore, NS1 inhibits NLRP3 inflammasome activation by targeting the ASC [294] and TRIM25 [197] (Figure 6).

IAV also antagonizes the NLRP3-mediated signaling via PB1-F2, which interacts with NLRP3 and interferes with the NLRP3 inflammasome activation [295,296,297] (Figure 6).

### 4.4. Antagonism of JAK-STAT Pathway

IAV antagonizes the JAK-STAT pathway by multiple mechanisms. Firstly, NS1 upregulates the expression of host proteins, suppressor of cytokine signaling (SOCS) 1 and 3 [298]; SOCS1 and SOCS3 possess the inherent ability to inhibit JAK-STAT pathway in IAV-infected cells by targeting the JAKs and STATs [299,300] (Figure 7). Secondly, IAV PB2 and membrane protein HA promote the degradation of JAK1 [301] and type I and II IFN receptors [302,303], respectively (Figure 7). Thirdly, IAV exploits host microRNAs to interfere with the JAK-STAT pathway (Figure 7). IAV downregulates the expression of miR-30, which suppresses SOCS1 and SOCS3 expression [304]. Furthermore, IAV employs miR-93 and put-miR-34 to downregulate the expression of JAK1 [305] and STAT3 [306], respectively (Figure 7).

### 4.5. Antagonism of ISGs

In addition to antagonizing the upstream innate immune pathways, IAV directly antagonizes some of the ISGs by multiple mechanisms, and here too, IAV employs NS1 protein. NS1 competes with ISGs, PKR, and OAS (Figure 7) for binding to viral dsRNA PAMP and blocks the activation of PKR-mediated pathway, which inhibits viral protein synthesis [307,308,309] and RNase L, which degrades viral RNA [310], respectively. Furthermore, NS1 interacts with hGBP-1 (Figure 7) and inhibits the GTPase activity required for its antiviral function [311]. To antagonize tetherin, IAV employs its membrane protein, M2 which interacts with tetherin (Figure 7) and promotes its degradation [312]. Finally, IAV recruits the ubiquitin ligase, NEDD4 [304,313] and methyltransferase, SET7 [314] (Figure 7) to ubiquitinate and methylate IFITM3, respectively, and decrease its antiviral activity.

## 5. IAV’s Escape from the Host Innate Immune Response

IAV can escape the innate antiviral response in hosts carrying the genetic variants of antiviral factors due to single-nucleotide polymorphisms (SNPs) or mutations (Table 1), which reduce their antiviral activity and weaken the innate immune response. Furthermore, IAV can escape the host restriction by acquiring mutations in viral targets of antiviral factors.

### 5.1. Escape from TLR- and RLR-Mediated Sensing

Multiple variants of TLR genes have been reported in humans. IAV escapes the TLR-mediated sensing and causes severe disease in humans carrying some of those variants [315,316,317,318,319,320]. IAV infection leads to pneumonia or acute respiratory distress syndrome (ARDS) in humans carrying the SNPs rs5743313/CT [315], rs5743313 [316], L412F [318], P554S/P680L [317,319], and rs3775291/rs3775290 [320] in TLR3 gene (Table 1).

Similarly, IAV escapes the RIG-I-mediated sensing and downstream signaling in humans carrying the SNPs p.R71H/p.P885S [321] and rs4487862 [322] in RIG-I (DDX58) gene and causes severe disease (Table 1).

### 5.2. Escape from IRF-Mediated Signaling

The variants of IRF7 and IRF9 genes have also been reported in humans [323,324,325,326,327]. IAV escapes the weakened IFN response and causes severe disease in humans carrying the IRF7 variants p.Phe410Val (F410V)/p.Gln421X(Q421X) [324] and E331V [326] and IRF9 variant c.991G > A [325] (Table 1).

### 5.3. Escape from ISG-Mediated Restriction

The ISG MxA is a GTPase and restricts IAV infection by sequestering the vRNPs [224]. IAV escapes the MxA-mediated restriction in humans carrying Mx1 gene variants, which encode for MxA deficient in GTPase activity [328,329,330] (Table 1). Human MxA is also a barrier against the transmission of zoonotic IAVs to humans [224]. However, human IAV have acquired the mutations in their NP to escape the MxA-mediated restriction in human cells [331,332,333]. Similarly, zoonotic IAV escapes the human MxA barrier by acquiring human-adaptive mutations in their NP [334,335,336,337].

IFITM3 is another prominent ISG that localizes to the endosome and restricts IAV infection by interfering with its endocytic entry to host cells [224]. IAV escapes the IFITM3 restriction and causes severe disease in humans [338,339], mainly from the Asian ethnicity [316,340,341,342,343,344,345,346,347], carrying the IFITM3 gene variant rs12252-C. This variant encodes N-terminally truncated IFITM3, which is unable to localize to the endosome, and hence, allows IAV to escape the IFITM3 restriction [338,348,349,350] (Table 1). Furthermore, avian IAVs may escape the IFITM3 restriction in human cells with ineffective endosomal acidification [351].

Similarly, IAV escapes the restriction imposed by ISGs, OAS, and Serpin 1 in humans carrying OAS1 gene variant rs10774671 [352] and Serpin 1 gene variant rs6092 [353], respectively (Table 1).

## 6. Summary

The host employs multiple innate immune pathways and a complex network of factors in each pathway to sense, block, and eliminate the IAV infection. However, IAV employs its own proteins, notably NS1, as well as recruits host factors, both proteins and ncRNAs, to antagonize multiple steps of the host innate immune response. 

IAV continues to pose a significant burden on global public health annually through seasonal epidemics [354]. Furthermore, the threat of another IAV pandemic is real as exemplified by the increased incidents of zoonotic infections, evolution, and cross-species transmission of avian IAVs, particularly the H5N1 subtype [12]. Alarmingly, an avian IAV H5N1 subtype has been discovered recently to infect dairy cows—a previously unknown host to IAV [355]—and associated dairy farm workers [356,357]. Three classes of antiviral drugs—M2 ion channel inhibitors, NA inhibitors, and PA inhibitors—are available to treat IAV infections. However, these drugs are prone to be ineffective over time because they target the IAV components M2, NA, and PA, respectively, and IAV can mutate these components to acquire resistance to these drugs [358,359]. Indeed, the M2 ion channel inhibitors are not recommended for the treatment of IAV infections anymore, because the majority of circulating IAV strains have acquired resistance to them [360]. Therefore, a comprehensive knowledge of the interplay between host innate immune response and IAV is crucial to designing alternative antiviral therapies targeting the host factors involved in innate immune response [361,362]. 

Indeed, some of the knowledge gained in this space has already been applied, e.g., the use of TLR agonists for treating IAV infections [363] or as adjuvants in flu vaccine formulations [364]. Furthermore, inhibitors of critical components of the innate pathways, e.g., RIPK3 in ZBP1-mediated necroptosis [365], are being developed to prevent the severity of IAV disease.

## Figures and Tables

**Table 1:** Host gene variants allowing IAV to escape the innate immune response.

Genes	Variants/Mutations	Effect
TLR3	rs5743313/CT, rs5743313, L412F, P554S/P680L, rs3775291/rs3775290	Escape from TLR3-mediated signaling
RIG-I (DDX58)	p.R71H/p.P885S, rs4487862	Escape from RIG-I-mediated signaling
IRF7	p.Phe410Val (F410V)/p.Gln421X(Q421X), E331V	Escape from weakened IFN response
IRF9	c.991G > A	Escape from weakened IFN response
MxA	Mutations in GTPase domain	Escape from MxA-mediated restriction
IFITM3	rs12252-C	Escape from IFITM3-mediated restriction
OAS	rs10774671	Escape from OAS-mediated restriction
Serpin 1	rs6092	Escape from Serpin 1-mediated restriction

## References

[1] Riera Romo M., Perez-Martinez D., Castillo Ferrer C. (2016). Innate immunity in vertebrates: An overview. Immunology.

[2] Beutler B. (2004). Innate immunity: An overview. Mol. Immunol..

[3] Iwama R.E., Moran Y. (2023). Origins and diversification of animal innate immune responses against viral infections. Nat. Ecol. Evol..

[4] Bergeron H.C., Hansen M.R., Tripp R.A. (2023). Interferons-Implications in the Immune Response to Respiratory Viruses. Microorganisms.

[5] Zhu J., Chiang C., Gack M.U. (2023). Viral evasion of the interferon response at a glance. J. Cell Sci..

[6] Wei L., Wang X., Zhou H. (2023). Interaction among inflammasome, PANoptosise, and innate immune cells in infection of influenza virus: Updated review. Immun. Inflamm. Dis..

[7] Stroz S., Kosiorek P., Stasiak-Barmuta A. (2024). The COVID-19 inflammation and high mortality mechanism trigger. Immunogenetics.

[8] King A.M.Q., Adams M.J., Carstens E.B., Lefkowitz E.J. (2012). Orthomyxoviridae. Virus Taxonomy.

[9] Su S., Fu X., Li G., Kerlin F., Veit M. (2017). Novel Influenza D virus: Epidemiology, pathology, evolution and biological characteristics. Virulence.

[10] Russell C.A., Fouchier R.A.M., Ghaswalla P., Park Y., Vicic N., Ananworanich J., Nachbagauer R., Rudin D. (2024). Seasonal influenza vaccine performance and the potential benefits of mRNA vaccines. Hum. Vaccin. Immunother..

[11] Long J.S., Mistry B., Haslam S.M., Barclay W.S. (2019). Host and viral determinants of influenza A virus species specificity. Nat. Rev. Microbiol..

[12] Kang M., Wang L.-F., Sun B.-W., Wan W.-B., Ji X., Baele G., Bi Y.-H., Suchard M.A., Lai A., Zhang M. (2024). Zoonotic infections by avian influenza virus: Changing global epidemiology, investigation, and control. Lancet Infect. Dis..

[13] Tang C.Y., Ramesh A., Wan X.-F., Tang Y.-W., Hindiyeh M.Y., Liu D., Sails A., Spearman P., Zhang J.-R. (2024). Avian and swine influenza viruses. Molecular Medical Microbiology.

[14] Dou D., Revol R., Ostbye H., Wang H., Daniels R. (2018). Influenza A Virus Cell Entry, Replication, Virion Assembly and Movement. Front. Immunol..

[15] Li H.C., Yang C.H., Lo S.Y. (2022). Strategies of Influenza A Virus to Ensure the Translation of Viral mRNAs. Pathogens.

[16] Anderson K.V., Jurgens G., Nusslein-Volhard C. (1985). Establishment of dorsal-ventral polarity in the Drosophila embryo: Genetic studies on the role of the Toll gene product. Cell.

[17] Chiang C., Beachy P.A. (1994). Expression of a novel Toll-like gene spans the parasegment boundary and contributes to hedgehog function in the adult eye of Drosophila. Mech. Dev..

[18] Lemaitre B., Nicolas E., Michaut L., Reichhart J.M., Hoffmann J.A. (1996). The dorsoventral regulatory gene cassette spatzle/Toll/cactus controls the potent antifungal response in Drosophila adults. Cell.

[19] Medzhitov R., Preston-Hurlburt P., Janeway C.A. (1997). A human homologue of the Drosophila Toll protein signals activation of adaptive immunity. Nature.

[20] Rock F.L., Hardiman G., Timans J.C., Kastelein R.A., Bazan J.F. (1998). A family of human receptors structurally related to Drosophila Toll. Proc. Natl. Acad. Sci. USA.

[21] Hoshino K., Takeuchi O., Kawai T., Sanjo H., Ogawa T., Takeda Y., Takeda K., Akira S. (1999). Cutting edge: Toll-like receptor 4 (TLR4)-deficient mice are hyporesponsive to lipopolysaccharide: Evidence for TLR4 as the Lps gene product. J. Immunol..

[22] Poltorak A., He X., Smirnova I., Liu M.Y., Van Huffel C., Du X., Birdwell D., Alejos E., Silva M., Galanos C. (1998). Defective LPS signaling in C3H/HeJ and C57BL/10ScCr mice: Mutations in Tlr4 gene. Science.

[23] Qureshi S.T., Lariviere L., Leveque G., Clermont S., Moore K.J., Gros P., Malo D. (1999). Endotoxin-tolerant mice have mutations in Toll-like receptor 4 (Tlr4). J. Exp. Med..

[24] Kirschning C.J., Wesche H., Merrill Ayres T., Rothe M. (1998). Human toll-like receptor 2 confers responsiveness to bacterial lipopolysaccharide. J. Exp. Med..

[25] Yang R.B., Mark M.R., Gray A., Huang A., Xie M.H., Zhang M., Goddard A., Wood W.I., Gurney A.L., Godowski P.J. (1998). Toll-like receptor-2 mediates lipopolysaccharide-induced cellular signalling. Nature.

[26] Kawasaki T., Kawai T. (2014). Toll-like receptor signaling pathways. Front. Immunol..

[27] Botos I., Segal D.M., Davies D.R. (2011). The structural biology of Toll-like receptors. Structure.

[28] Lester S.N., Li K. (2014). Toll-like receptors in antiviral innate immunity. J. Mol. Biol..

[29] Guillot L., Le Goffic R., Bloch S., Escriou N., Akira S., Chignard M., Si-Tahar M. (2005). Involvement of toll-like receptor 3 in the immune response of lung epithelial cells to double-stranded RNA and influenza A virus. J. Biol. Chem..

[30] Le Goffic R., Balloy V., Lagranderie M., Alexopoulou L., Escriou N., Flavell R., Chignard M., Si-Tahar M. (2006). Detrimental contribution of the Toll-like receptor (TLR)3 to influenza A virus-induced acute pneumonia. PLoS Pathog..

[31] Diebold S.S., Kaisho T., Hemmi H., Akira S., Reis e Sousa C. (2004). Innate antiviral responses by means of TLR7-mediated recognition of single-stranded RNA. Science.

[32] Lund J.M., Alexopoulou L., Sato A., Karow M., Adams N.C., Gale N.W., Iwasaki A., Flavell R.A. (2004). Recognition of single-stranded RNA viruses by Toll-like receptor 7. Proc. Natl. Acad. Sci. USA.

[33] Wang J.P., Bowen G.N., Padden C., Cerny A., Finberg R.W., Newburger P.E., Kurt-Jones E.A. (2008). Toll-like receptor-mediated activation of neutrophils by influenza A virus. Blood.

[34] Le Goffic R., Pothlichet J., Vitour D., Fujita T., Meurs E., Chignard M., Si-Tahar M. (2007). Cutting Edge: Influenza A virus activates TLR3-dependent inflammatory and RIG-I-dependent antiviral responses in human lung epithelial cells. J. Immunol..

[35] Tsai S.Y., Segovia J.A., Chang T.H., Morris I.R., Berton M.T., Tessier P.A., Tardif M.R., Cesaro A., Bose S. (2014). DAMP molecule S100A9 acts as a molecular pattern to enhance inflammation during influenza A virus infection: Role of DDX21-TRIF-TLR4-MyD88 pathway. PLoS Pathog..

[36] Imai Y., Kuba K., Neely G.G., Yaghubian-Malhami R., Perkmann T., van Loo G., Ermolaeva M., Veldhuizen R., Leung Y.H., Wang H. (2008). Identification of oxidative stress and Toll-like receptor 4 signaling as a key pathway of acute lung injury. Cell.

[37] Lee S.M., Kok K.H., Jaume M., Cheung T.K., Yip T.F., Lai J.C., Guan Y., Webster R.G., Jin D.Y., Peiris J.S. (2014). Toll-like receptor 10 is involved in induction of innate immune responses to influenza virus infection. Proc. Natl. Acad. Sci. USA.

[38] Rehwinkel J., Gack M.U. (2020). RIG-I-like receptors: Their regulation and roles in RNA sensing. Nat. Rev. Immunol..

[39] Sun Y.W. (1997). RIG-I, a Human Homolog Gene of RNA Helicase, Is Induced by Retinoic Acid during the Differentiation of Acute Promyelocytic Luekemia Cell. Thesis.

[40] Liu T.X., Zhang J.W., Tao J., Zhang R.B., Zhang Q.H., Zhao C.J., Tong J.H., Lanotte M., Waxman S., Chen S.J. (2000). Gene expression networks underlying retinoic acid-induced differentiation of acute promyelocytic leukemia cells. Blood.

[41] Imaizumi T., Aratani S., Nakajima T., Carlson M., Matsumiya T., Tanji K., Ookawa K., Yoshida H., Tsuchida S., McIntyre T.M. (2002). Retinoic acid-inducible gene-I is induced in endothelial cells by LPS and regulates expression of COX-2. Biochem. Biophys. Res. Commun..

[42] Zhang X., Wang C., Schook L.B., Hawken R.J., Rutherford M.S. (2000). An RNA helicase, RHIV -1, induced by porcine reproductive and respiratory syndrome virus (PRRSV) is mapped on porcine chromosome 10q13. Microb. Pathog..

[43] Yoneyama M., Kikuchi M., Natsukawa T., Shinobu N., Imaizumi T., Miyagishi M., Taira K., Akira S., Fujita T. (2004). The RNA helicase RIG-I has an essential function in double-stranded RNA-induced innate antiviral responses. Nat. Immunol..

[44] Kato H., Sato S., Yoneyama M., Yamamoto M., Uematsu S., Matsui K., Tsujimura T., Takeda K., Fujita T., Takeuchi O. (2005). Cell type-specific involvement of RIG-I in antiviral response. Immunity.

[45] Kato H., Takeuchi O., Sato S., Yoneyama M., Yamamoto M., Matsui K., Uematsu S., Jung A., Kawai T., Ishii K.J. (2006). Differential roles of MDA5 and RIG-I helicases in the recognition of RNA viruses. Nature.

[46] Hornung V., Ellegast J., Kim S., Brzozka K., Jung A., Kato H., Poeck H., Akira S., Conzelmann K.K., Schlee M. (2006). 5′-Triphosphate RNA is the ligand for RIG-I. Science.

[47] Pichlmair A., Schulz O., Tan C.P., Naslund T.I., Liljestrom P., Weber F., Reis e Sousa C. (2006). RIG-I-mediated antiviral responses to single-stranded RNA bearing 5′-phosphates. Science.

[48] Goubau D., Schlee M., Deddouche S., Pruijssers A.J., Zillinger T., Goldeck M., Schuberth C., Van der Veen A.G., Fujimura T., Rehwinkel J. (2014). Antiviral immunity via RIG-I-mediated recognition of RNA bearing 5′-diphosphates. Nature.

[49] Schlee M., Roth A., Hornung V., Hagmann C.A., Wimmenauer V., Barchet W., Coch C., Janke M., Mihailovic A., Wardle G. (2009). Recognition of 5′ triphosphate by RIG-I helicase requires short blunt double-stranded RNA as contained in panhandle of negative-strand virus. Immunity.

[50] Schmidt A., Schwerd T., Hamm W., Hellmuth J.C., Cui S., Wenzel M., Hoffmann F.S., Michallet M.C., Besch R., Hopfner K.P. (2009). 5′-triphosphate RNA requires base-paired structures to activate antiviral signaling via RIG-I. Proc. Natl. Acad. Sci. USA.

[51] Kato H., Takeuchi O., Mikamo-Satoh E., Hirai R., Kawai T., Matsushita K., Hiiragi A., Dermody T.S., Fujita T., Akira S. (2008). Length-dependent recognition of double-stranded ribonucleic acids by retinoic acid-inducible gene-I and melanoma differentiation-associated gene 5. J. Exp. Med..

[52] Baum A., Sachidanandam R., Garcia-Sastre A. (2010). Preference of RIG-I for short viral RNA molecules in infected cells revealed by next-generation sequencing. Proc. Natl. Acad. Sci. USA.

[53] Rehwinkel J., Tan C.P., Goubau D., Schulz O., Pichlmair A., Bier K., Robb N., Vreede F., Barclay W., Fodor E. (2010). RIG-I detects viral genomic RNA during negative-strand RNA virus infection. Cell.

[54] Yin X., Riva L., Pu Y., Martin-Sancho L., Kanamune J., Yamamoto Y., Sakai K., Gotoh S., Miorin L., De Jesus P.D. (2021). MDA5 Governs the Innate Immune Response to SARS-CoV-2 in Lung Epithelial Cells. Cell Rep..

[55] Liu G., Park H.S., Pyo H.M., Liu Q., Zhou Y. (2015). Influenza A Virus Panhandle Structure Is Directly Involved in RIG-I Activation and Interferon Induction. J. Virol..

[56] Te Velthuis A.J.W., Long J.C., Bauer D.L.V., Fan R.L.Y., Yen H.L., Sharps J., Siegers J.Y., Killip M.J., French H., Oliva-Martin M.J. (2018). Mini viral RNAs act as innate immune agonists during influenza virus infection. Nat. Microbiol..

[57] Lee M.K., Kim H.E., Park E.B., Lee J., Kim K.H., Lim K., Yum S., Lee Y.H., Kang S.J., Lee J.H. (2016). Structural features of influenza A virus panhandle RNA enabling the activation of RIG-I independently of 5′-triphosphate. Nucleic Acids Res..

[58] Liu G., Lu Y., Thulasi Raman S.N., Xu F., Wu Q., Li Z., Brownlie R., Liu Q., Zhou Y. (2018). Nuclear-resident RIG-I senses viral replication inducing antiviral immunity. Nat. Commun..

[59] Fu Y., Comella N., Tognazzi K., Brown L.F., Dvorak H.F., Kocher O. (1999). Cloning of DLM-1, a novel gene that is up-regulated in activated macrophages, using RNA differential display. Gene.

[60] Schwartz T., Behlke J., Lowenhaupt K., Heinemann U., Rich A. (2001). Structure of the DLM-1-Z-DNA complex reveals a conserved family of Z-DNA-binding proteins. Nat. Struct. Biol..

[61] Ha S.C., Van Quyen D., Hwang H.Y., Oh D.B., Brown B.A., Lee S.M., Park H.J., Ahn J.H., Kim K.K., Kim Y.G. (2006). Biochemical characterization and preliminary X-ray crystallographic study of the domains of human ZBP1 bound to left-handed Z-DNA. Biochim. Biophys. Acta.

[62] Deigendesch N., Koch-Nolte F., Rothenburg S. (2006). ZBP1 subcellular localization and association with stress granules is controlled by its Z-DNA binding domains. Nucleic Acids Res..

[63] Takaoka A., Wang Z., Choi M.K., Yanai H., Negishi H., Ban T., Lu Y., Miyagishi M., Kodama T., Honda K. (2007). DAI (DLM-1/ZBP1) is a cytosolic DNA sensor and an activator of innate immune response. Nature.

[64] Rebsamen M., Heinz L.X., Meylan E., Michallet M.C., Schroder K., Hofmann K., Vazquez J., Benedict C.A., Tschopp J. (2009). DAI/ZBP1 recruits RIP1 and RIP3 through RIP homotypic interaction motifs to activate NF-kappaB. EMBO Rep..

[65] Maelfait J., Liverpool L., Bridgeman A., Ragan K.B., Upton J.W., Rehwinkel J. (2017). Sensing of viral and endogenous RNA by ZBP1/DAI induces necroptosis. EMBO J..

[66] Ha S.C., Kim D., Hwang H.Y., Rich A., Kim Y.G., Kim K.K. (2008). The crystal structure of the second Z-DNA binding domain of human DAI (ZBP1) in complex with Z-DNA reveals an unusual binding mode to Z-DNA. Proc. Natl. Acad. Sci. USA.

[67] Song Q., Fan Y., Zhang H., Wang N. (2024). Z-DNA binding protein 1 orchestrates innate immunity and inflammatory cell death. Cytokine Growth Factor Rev..

[68] Pham H.T., Park M.Y., Kim K.K., Kim Y.G., Ahn J.H. (2006). Intracellular localization of human ZBP1: Differential regulation by the Z-DNA binding domain, Zalpha, in splice variants. Biochem. Biophys. Res. Commun..

[69] Kuriakose T., Man S.M., Malireddi R.K., Karki R., Kesavardhana S., Place D.E., Neale G., Vogel P., Kanneganti T.D. (2016). ZBP1/DAI is an innate sensor of influenza virus triggering the NLRP3 inflammasome and programmed cell death pathways. Sci. Immunol..

[70] Zhang T., Yin C., Boyd D.F., Quarato G., Ingram J.P., Shubina M., Ragan K.B., Ishizuka T., Crawford J.C., Tummers B. (2020). Influenza Virus Z-RNAs Induce ZBP1-Mediated Necroptosis. Cell.

[71] Thapa R.J., Ingram J.P., Ragan K.B., Nogusa S., Boyd D.F., Benitez A.A., Sridharan H., Kosoff R., Shubina M., Landsteiner V.J. (2016). DAI Senses Influenza A Virus Genomic RNA and Activates RIPK3-Dependent Cell Death. Cell Host Microbe.

[72] Kesavardhana S., Kuriakose T., Guy C.S., Samir P., Malireddi R.K.S., Mishra A., Kanneganti T.D. (2017). ZBP1/DAI ubiquitination and sensing of influenza vRNPs activate programmed cell death. J. Exp. Med..

[73] Kesavardhana S., Malireddi R.K.S., Burton A.R., Porter S.N., Vogel P., Pruett-Miller S.M., Kanneganti T.D. (2020). The Zalpha2 domain of ZBP1 is a molecular switch regulating influenza-induced PANoptosis and perinatal lethality during development. J. Biol. Chem..

[74] Steimle V., Otten L.A., Zufferey M., Mach B. (1993). Complementation cloning of an MHC class II transactivator mutated in hereditary MHC class II deficiency (or bare lymphocyte syndrome). Cell.

[75] Harton J.A., Linhoff M.W., Zhang J., Ting J.P. (2002). Cutting edge: CATERPILLER: A large family of mammalian genes containing CARD, pyrin, nucleotide-binding, and leucine-rich repeat domains. J. Immunol..

[76] Schroder K., Tschopp J. (2010). The inflammasomes. Cell.

[77] Blevins H.M., Xu Y., Biby S., Zhang S. (2022). The NLRP3 Inflammasome Pathway: A Review of Mechanisms and Inhibitors for the Treatment of Inflammatory Diseases. Front. Aging Neurosci..

[78] Allen I.C., Scull M.A., Moore C.B., Holl E.K., McElvania-TeKippe E., Taxman D.J., Guthrie E.H., Pickles R.J., Ting J.P. (2009). The NLRP3 inflammasome mediates in vivo innate immunity to influenza A virus through recognition of viral RNA. Immunity.

[79] Ichinohe T., Pang I.K., Iwasaki A. (2010). Influenza virus activates inflammasomes via its intracellular M2 ion channel. Nat. Immunol..

[80] Pandey K.P., Zhou Y. (2022). Influenza A Virus Infection Activates NLRP3 Inflammasome through Trans-Golgi Network Dispersion. Viruses.

[81] McAuley J.L., Tate M.D., MacKenzie-Kludas C.J., Pinar A., Zeng W., Stutz A., Latz E., Brown L.E., Mansell A. (2013). Activation of the NLRP3 inflammasome by IAV virulence protein PB1-F2 contributes to severe pathophysiology and disease. PLoS Pathog..

[82] Pinar A., Dowling J.K., Bitto N.J., Robertson A.A., Latz E., Stewart C.R., Drummond G.R., Cooper M.A., McAuley J.L., Tate M.D. (2017). PB1-F2 Peptide Derived from Avian Influenza A Virus H7N9 Induces Inflammation via Activation of the NLRP3 Inflammasome. J. Biol. Chem..

[83] Lee S., Ishitsuka A., Noguchi M., Hirohama M., Fujiyasu Y., Petric P.P., Schwemmle M., Staeheli P., Nagata K., Kawaguchi A. (2019). Influenza restriction factor MxA functions as inflammasome sensor in the respiratory epithelium. Sci. Immunol..

[84] Motshwene P.G., Moncrieffe M.C., Grossmann J.G., Kao C., Ayaluru M., Sandercock A.M., Robinson C.V., Latz E., Gay N.J. (2009). An oligomeric signaling platform formed by the Toll-like receptor signal transducers MyD88 and IRAK-4. J. Biol. Chem..

[85] Vyncke L., Bovijn C., Pauwels E., Van Acker T., Ruyssinck E., Burg E., Tavernier J., Peelman F. (2016). Reconstructing the TIR Side of the Myddosome: A Paradigm for TIR-TIR Interactions. Structure.

[86] Kawagoe T., Sato S., Matsushita K., Kato H., Matsui K., Kumagai Y., Saitoh T., Kawai T., Takeuchi O., Akira S. (2008). Sequential control of Toll-like receptor-dependent responses by IRAK1 and IRAK2. Nat. Immunol..

[87] Li S., Strelow A., Fontana E.J., Wesche H. (2002). IRAK-4: A novel member of the IRAK family with the properties of an IRAK-kinase. Proc. Natl. Acad. Sci. USA.

[88] Wang C., Deng L., Hong M., Akkaraju G.R., Inoue J., Chen Z.J. (2001). TAK1 is a ubiquitin-dependent kinase of MKK and IKK. Nature.

[89] Wu C.J., Conze D.B., Li T., Srinivasula S.M., Ashwell J.D. (2006). Sensing of Lys 63-linked polyubiquitination by NEMO is a key event in NF-kappaB activation [corrected]. Nat. Cell Biol..

[90] Napetschnig J., Wu H. (2013). Molecular basis of NF-kappaB signaling. Annu. Rev. Biophys..

[91] Kawai T., Sato S., Ishii K.J., Coban C., Hemmi H., Yamamoto M., Terai K., Matsuda M., Inoue J., Uematsu S. (2004). Interferon-alpha induction through Toll-like receptors involves a direct interaction of IRF7 with MyD88 and TRAF6. Nat. Immunol..

[92] Schoenemeyer A., Barnes B.J., Mancl M.E., Latz E., Goutagny N., Pitha P.M., Fitzgerald K.A., Golenbock D.T. (2005). The interferon regulatory factor, IRF5, is a central mediator of toll-like receptor 7 signaling. J. Biol. Chem..

[93] Takaoka A., Yanai H., Kondo S., Duncan G., Negishi H., Mizutani T., Kano S., Honda K., Ohba Y., Mak T.W. (2005). Integral role of IRF-5 in the gene induction programme activated by Toll-like receptors. Nature.

[94] Bergstrom B., Aune M.H., Awuh J.A., Kojen J.F., Blix K.J., Ryan L., Flo T.H., Mollnes T.E., Espevik T., Stenvik J. (2015). TLR8 Senses Staphylococcus aureus RNA in Human Primary Monocytes and Macrophages and Induces IFN-beta Production via a TAK1-IKKbeta-IRF5 Signaling Pathway. J. Immunol..

[95] Alexopoulou L., Holt A.C., Medzhitov R., Flavell R.A. (2001). Recognition of double-stranded RNA and activation of NF-kappaB by Toll-like receptor 3. Nature.

[96] Hoebe K., Du X., Georgel P., Janssen E., Tabeta K., Kim S.O., Goode J., Lin P., Mann N., Mudd S. (2003). Identification of Lps2 as a key transducer of MyD88-independent TIR signalling. Nature.

[97] Oshiumi H., Matsumoto M., Funami K., Akazawa T., Seya T. (2003). TICAM-1, an adaptor molecule that participates in Toll-like receptor 3-mediated interferon-beta induction. Nat. Immunol..

[98] Yamamoto M., Sato S., Hemmi H., Hoshino K., Kaisho T., Sanjo H., Takeuchi O., Sugiyama M., Okabe M., Takeda K. (2003). Role of adaptor TRIF in the MyD88-independent toll-like receptor signaling pathway. Science.

[99] Cusson-Hermance N., Khurana S., Lee T.H., Fitzgerald K.A., Kelliher M.A. (2005). Rip1 mediates the Trif-dependent toll-like receptor 3- and 4-induced NF-kappaB activation but does not contribute to interferon regulatory factor 3 activation. J. Biol. Chem..

[100] Meylan E., Burns K., Hofmann K., Blancheteau V., Martinon F., Kelliher M., Tschopp J. (2004). RIP1 is an essential mediator of Toll-like receptor 3-induced NF-kappa B activation. Nat. Immunol..

[101] Chen F., Chen L., Li Y., Sang H., Zhang C., Yuan S., Yang J. (2022). TRAF3 Positively Regulates Host Innate Immune Resistance to Influenza A Virus Infection. Front. Cell. Infect. Microbiol..

[102] Hacker H., Redecke V., Blagoev B., Kratchmarova I., Hsu L.C., Wang G.G., Kamps M.P., Raz E., Wagner H., Hacker G. (2006). Specificity in Toll-like receptor signalling through distinct effector functions of TRAF3 and TRAF6. Nature.

[103] Oganesyan G., Saha S.K., Guo B., He J.Q., Shahangian A., Zarnegar B., Perry A., Cheng G. (2006). Critical role of TRAF3 in the Toll-like receptor-dependent and -independent antiviral response. Nature.

[104] Malik G., Zhou Y. (2020). Innate Immune Sensing of Influenza A Virus. Viruses.

[105] Seo S.U., Kwon H.J., Song J.H., Byun Y.H., Seong B.L., Kawai T., Akira S., Kweon M.N. (2010). MyD88 signaling is indispensable for primary influenza A virus infection but dispensable for secondary infection. J. Virol..

[106] Wu W., Zhang W., Duggan E.S., Booth J.L., Zou M.H., Metcalf J.P. (2015). RIG-I and TLR3 are both required for maximum interferon induction by influenza virus in human lung alveolar epithelial cells. Virology.

[107] Huo C., Jin Y., Zou S., Qi P., Xiao J., Tian H., Wang M., Hu Y. (2018). Lethal influenza A virus preferentially activates TLR3 and triggers a severe inflammatory response. Virus Res..

[108] Yong Y.H., Liu S.F., Hua G.H., Jia R.M., Gooneratne R., Zhao Y.T., Liao M., Ju X.H. (2018). Goose toll-like receptor 3 (TLR3) mediated IFN-gamma and IL-6 in anti-H5N1 avian influenza virus response. Vet. Immunol. Immunopathol..

[109] Park W.J., Han S.H., Kim D.H., Song Y.J., Lee J.B., Park S.Y., Song C.S., Lee S.W., Choi I.S. (2021). Induction of IFN-beta through TLR-3- and RIG-I-Mediated Signaling Pathways in Canine Respiratory Epithelial Cells Infected with H3N2 Canine Influenza Virus. J. Microbiol. Biotechnol..

[110] de Marcken M., Dhaliwal K., Danielsen A.C., Gautron A.S., Dominguez-Villar M. (2019). TLR7 and TLR8 activate distinct pathways in monocytes during RNA virus infection. Sci. Signal..

[111] Wies E., Wang M.K., Maharaj N.P., Chen K., Zhou S., Finberg R.W., Gack M.U. (2013). Dephosphorylation of the RNA sensors RIG-I and MDA5 by the phosphatase PP1 is essential for innate immune signaling. Immunity.

[112] Nistal-Villan E., Gack M.U., Martinez-Delgado G., Maharaj N.P., Inn K.S., Yang H., Wang R., Aggarwal A.K., Jung J.U., Garcia-Sastre A. (2010). Negative role of RIG-I serine 8 phosphorylation in the regulation of interferon-beta production. J. Biol. Chem..

[113] Gack M.U., Nistal-Villan E., Inn K.S., Garcia-Sastre A., Jung J.U. (2010). Phosphorylation-mediated negative regulation of RIG-I antiviral activity. J. Virol..

[114] Sun Z., Ren H., Liu Y., Teeling J.L., Gu J. (2011). Phosphorylation of RIG-I by casein kinase II inhibits its antiviral response. J. Virol..

[115] Maharaj N.P., Wies E., Stoll A., Gack M.U. (2012). Conventional protein kinase C-alpha (PKC-alpha) and PKC-beta negatively regulate RIG-I antiviral signal transduction. J. Virol..

[116] Kowalinski E., Lunardi T., McCarthy A.A., Louber J., Brunel J., Grigorov B., Gerlier D., Cusack S. (2011). Structural basis for the activation of innate immune pattern-recognition receptor RIG-I by viral RNA. Cell.

[117] Gee P., Chua P.K., Gevorkyan J., Klumpp K., Najera I., Swinney D.C., Deval J. (2008). Essential role of the N-terminal domain in the regulation of RIG-I ATPase activity. J. Biol. Chem..

[118] Jiang F., Ramanathan A., Miller M.T., Tang G.Q., Gale M., Patel S.S., Marcotrigiano J. (2011). Structural basis of RNA recognition and activation by innate immune receptor RIG-I. Nature.

[119] Cui S., Eisenacher K., Kirchhofer A., Brzozka K., Lammens A., Lammens K., Fujita T., Conzelmann K.K., Krug A., Hopfner K.P. (2008). The C-terminal regulatory domain is the RNA 5′-triphosphate sensor of RIG-I. Mol. Cell.

[120] Luo D., Ding S.C., Vela A., Kohlway A., Lindenbach B.D., Pyle A.M. (2011). Structural insights into RNA recognition by RIG-I. Cell.

[121] Peisley A., Wu B., Yao H., Walz T., Hur S. (2013). RIG-I forms signaling-competent filaments in an ATP-dependent, ubiquitin-independent manner. Mol. Cell.

[122] Luo D., Kohlway A., Vela A., Pyle A.M. (2012). Visualizing the determinants of viral RNA recognition by innate immune sensor RIG-I. Structure.

[123] Gao D., Yang Y.K., Wang R.P., Zhou X., Diao F.C., Li M.D., Zhai Z.H., Jiang Z.F., Chen D.Y. (2009). REUL is a novel E3 ubiquitin ligase and stimulator of retinoic-acid-inducible gene-I. PLoS ONE.

[124] Oshiumi H., Matsumoto M., Hatakeyama S., Seya T. (2009). Riplet/RNF135, a RING finger protein, ubiquitinates RIG-I to promote interferon-beta induction during the early phase of viral infection. J. Biol. Chem..

[125] Oshiumi H., Miyashita M., Inoue N., Okabe M., Matsumoto M., Seya T. (2010). The ubiquitin ligase Riplet is essential for RIG-I-dependent innate immune responses to RNA virus infection. Cell Host Microbe.

[126] Gack M.U., Shin Y.C., Joo C.H., Urano T., Liang C., Sun L., Takeuchi O., Akira S., Chen Z., Inoue S. (2007). TRIM25 RING-finger E3 ubiquitin ligase is essential for RIG-I-mediated antiviral activity. Nature.

[127] Zeng W., Sun L., Jiang X., Chen X., Hou F., Adhikari A., Xu M., Chen Z.J. (2010). Reconstitution of the RIG-I pathway reveals a signaling role of unanchored polyubiquitin chains in innate immunity. Cell.

[128] Xian H., Xie W., Yang S., Liu Q., Xia X., Jin S., Sun T., Cui J. (2017). Stratified ubiquitination of RIG-I creates robust immune response and induces selective gene expression. Sci. Adv..

[129] Hayman T.J., Hsu A.C., Kolesnik T.B., Dagley L.F., Willemsen J., Tate M.D., Baker P.J., Kershaw N.J., Kedzierski L., Webb A.I. (2019). RIPLET, and not TRIM25, is required for endogenous RIG-I-dependent antiviral responses. Immunol. Cell Biol..

[130] Jiang X., Kinch L.N., Brautigam C.A., Chen X., Du F., Grishin N.V., Chen Z.J. (2012). Ubiquitin-induced oligomerization of the RNA sensors RIG-I and MDA5 activates antiviral innate immune response. Immunity.

[131] Liu H.M., Loo Y.M., Horner S.M., Zornetzer G.A., Katze M.G., Gale M. (2012). The mitochondrial targeting chaperone 14-3-3epsilon regulates a RIG-I translocon that mediates membrane association and innate antiviral immunity. Cell Host Microbe.

[132] Horner S.M., Liu H.M., Park H.S., Briley J., Gale M. (2011). Mitochondrial-associated endoplasmic reticulum membranes (MAM) form innate immune synapses and are targeted by hepatitis C virus. Proc. Natl. Acad. Sci. USA.

[133] Seth R.B., Sun L., Ea C.K., Chen Z.J. (2005). Identification and characterization of MAVS, a mitochondrial antiviral signaling protein that activates NF-kappaB and IRF 3. Cell.

[134] Dixit E., Boulant S., Zhang Y., Lee A.S., Odendall C., Shum B., Hacohen N., Chen Z.J., Whelan S.P., Fransen M. (2010). Peroxisomes are signaling platforms for antiviral innate immunity. Cell.

[135] Hou F., Sun L., Zheng H., Skaug B., Jiang Q.X., Chen Z.J. (2011). MAVS forms functional prion-like aggregates to activate and propagate antiviral innate immune response. Cell.

[136] Michallet M.C., Meylan E., Ermolaeva M.A., Vazquez J., Rebsamen M., Curran J., Poeck H., Bscheider M., Hartmann G., Konig M. (2008). TRADD protein is an essential component of the RIG-like helicase antiviral pathway. Immunity.

[137] Guo B., Cheng G. (2007). Modulation of the interferon antiviral response by the TBK1/IKKi adaptor protein TANK. J. Biol. Chem..

[138] Zhao T., Yang L., Sun Q., Arguello M., Ballard D.W., Hiscott J., Lin R. (2007). The NEMO adaptor bridges the nuclear factor-kappaB and interferon regulatory factor signaling pathways. Nat. Immunol..

[139] Pippig D.A., Hellmuth J.C., Cui S., Kirchhofer A., Lammens K., Lammens A., Schmidt A., Rothenfusser S., Hopfner K.P. (2009). The regulatory domain of the RIG-I family ATPase LGP2 senses double-stranded RNA. Nucleic Acids Res..

[140] Li X., Ranjith-Kumar C.T., Brooks M.T., Dharmaiah S., Herr A.B., Kao C., Li P. (2009). The RIG-I-like receptor LGP2 recognizes the termini of double-stranded RNA. J. Biol. Chem..

[141] Rothenfusser S., Goutagny N., DiPerna G., Gong M., Monks B.G., Schoenemeyer A., Yamamoto M., Akira S., Fitzgerald K.A. (2005). The RNA helicase Lgp2 inhibits TLR-independent sensing of viral replication by retinoic acid-inducible gene-I. J. Immunol..

[142] Rodriguez K.R., Bruns A.M., Horvath C.M. (2014). MDA5 and LGP2: Accomplices and antagonists of antiviral signal transduction. J. Virol..

[143] Childs K.S., Randall R.E., Goodbourn S. (2013). LGP2 plays a critical role in sensitizing mda-5 to activation by double-stranded RNA. PLoS ONE.

[144] Uchikawa E., Lethier M., Malet H., Brunel J., Gerlier D., Cusack S. (2016). Structural Analysis of dsRNA Binding to Anti-viral Pattern Recognition Receptors LGP2 and MDA5. Mol. Cell.

[145] Bruns A.M., Leser G.P., Lamb R.A., Horvath C.M. (2014). The innate immune sensor LGP2 activates antiviral signaling by regulating MDA5-RNA interaction and filament assembly. Mol. Cell.

[146] Duic I., Tadakuma H., Harada Y., Yamaue R., Deguchi K., Suzuki Y., Yoshimura S.H., Kato H., Takeyasu K., Fujita T. (2020). Viral RNA recognition by LGP2 and MDA5, and activation of signaling through step-by-step conformational changes. Nucleic Acids Res..

[147] Bruns A.M., Pollpeter D., Hadizadeh N., Myong S., Marko J.F., Horvath C.M. (2013). ATP hydrolysis enhances RNA recognition and antiviral signal transduction by the innate immune sensor, laboratory of genetics and physiology 2 (LGP2). J. Biol. Chem..

[148] Singh R., Wu Y., Herrero Del Valle A., Leigh K.E., Mong S., Cheng M.T.K., Ferguson B.J., Modis Y. (2024). Contrasting functions of ATP hydrolysis by MDA5 and LGP2 in viral RNA sensing. J. Biol. Chem..

[149] Opitz B., Rejaibi A., Dauber B., Eckhard J., Vinzing M., Schmeck B., Hippenstiel S., Suttorp N., Wolff T. (2007). IFNbeta induction by influenza A virus is mediated by RIG-I which is regulated by the viral NS1 protein. Cell Microbiol..

[150] Kandasamy M., Suryawanshi A., Tundup S., Perez J.T., Schmolke M., Manicassamy S., Manicassamy B. (2016). RIG-I Signaling Is Critical for Efficient Polyfunctional T Cell Responses during Influenza Virus Infection. PLoS Pathog..

[151] Hao W., Wang L., Li S. (2020). FKBP5 Regulates RIG-I-Mediated NF-kappaB Activation and Influenza A Virus Infection. Viruses.

[152] Sun N., Jiang L., Ye M., Wang Y., Wang G., Wan X., Zhao Y., Wen X., Liang L., Ma S. (2020). TRIM35 mediates protection against influenza infection by activating TRAF3 and degrading viral PB2. Protein Cell.

[153] Mohamed A.A., Soler S., Wegner J., Bartok E., Stankovic S., Brooks A.G., Schlee M. (2023). Influenza A Infection Stimulates RIG-I and Enhances Effector Function of Primary Human NK Cells. Int. J. Mol. Sci..

[154] Barber M.R., Aldridge J.R., Fleming-Canepa X., Wang Y.D., Webster R.G., Magor K.E. (2013). Identification of avian RIG-I responsive genes during influenza infection. Mol. Immunol..

[155] Blaine A.H., Miranzo-Navarro D., Campbell L.K., Aldridge J.R., Webster R.G., Magor K.E. (2015). Duck TRIM27-L enhances MAVS signaling and is absent in chickens and turkeys. Mol. Immunol..

[156] Shao Q., Xu W., Guo Q., Yan L., Rui L., Liu J., Zhao Y., Li Z. (2015). RIG-I from waterfowl and mammals differ in their abilities to induce antiviral responses against influenza A viruses. J. Gen. Virol..

[157] Wang Z., Ye S., Yao C., Wang J., Mao J., Xu L., Liu Y., Fu C., Lu G., Li S. (2021). Antiviral Activity of Canine RIG-I against Canine Influenza Virus and Interactions between Canine RIG-I and CIV. Viruses.

[158] Zhai B., Liu L., Li X., Lv X., Wu J., Li J., Lin S., Yin Y., Lan J., Du J. (2022). The Variation of Duck RIG-I-Mediated Innate Immune Response Induced by Different Virulence Avian Influenza Viruses. Front. Microbiol..

[159] Barber M.R., Aldridge J.R., Webster R.G., Magor K.E. (2010). Association of RIG-I with innate immunity of ducks to influenza. Proc. Natl. Acad. Sci. USA.

[160] Wei Y., Zeng Y., Zhang X., Xu S., Wang Z., Du Y., Zhang B., Lei C.Q., Zhu Q. (2020). The Nucleoprotein of H7N9 Influenza Virus Positively Regulates TRAF3-Mediated Innate Signaling and Attenuates Viral Virulence in Mice. J. Virol..

[161] Ranjan P., Singh N., Kumar A., Neerincx A., Kremmer E., Cao W., Davis W.G., Katz J.M., Gangappa S., Lin R. (2015). NLRC5 interacts with RIG-I to induce a robust antiviral response against influenza virus infection. Eur. J. Immunol..

[162] Zhao L., Zhu J., Zhou H., Zhao Z., Zou Z., Liu X., Lin X., Zhang X., Deng X., Wang R. (2015). Identification of cellular microRNA-136 as a dual regulator of RIG-I-mediated innate immunity that antagonizes H5N1 IAV replication in A549 cells. Sci. Rep..

[163] Song Y., Lai L., Chong Z., He J., Zhang Y., Xue Y., Xie Y., Chen S., Dong P., Chen L. (2017). E3 ligase FBXW7 is critical for RIG-I stabilization during antiviral responses. Nat. Commun..

[164] Jahan A.S., Biquand E., Munoz-Moreno R., Le Quang A., Mok C.K., Wong H.H., Teo Q.W., Valkenburg S.A., Chin A.W.H., Man Poon L.L. (2020). OTUB1 Is a Key Regulator of RIG-I-Dependent Immune Signaling and Is Targeted for Proteasomal Degradation by Influenza A NS1. Cell Rep..

[165] Nunez R.D., Budt M., Saenger S., Paki K., Arnold U., Sadewasser A., Wolff T. (2018). The RNA Helicase DDX6 Associates with RIG-I to Augment Induction of Antiviral Signaling. Int. J. Mol. Sci..

[166] Marcos-Villar L., Nistal-Villan E., Zamarreno N., Garaigorta U., Gastaminza P., Nieto A. (2020). Interferon-β Stimulation Elicited by the Influenza Virus Is Regulated by the Histone Methylase Dot1L through the RIG-I-TRIM25 Signaling Axis. Cells.

[167] Jiang Z., Wei F., Zhang Y., Wang T., Gao W., Yu S., Sun H., Pu J., Sun Y., Wang M. (2021). IFI16 directly senses viral RNA and enhances RIG-I transcription and activation to restrict influenza virus infection. Nat. Microbiol..

[168] Steinberg J., Wadenpohl T., Jung S. (2021). The Endogenous RIG-I Ligand Is Generated in Influenza A-Virus Infected Cells. Viruses.

[169] Acharya D., Reis R., Volcic M., Liu G., Wang M.K., Chia B.S., Nchioua R., Gross R., Munch J., Kirchhoff F. (2022). Actin cytoskeleton remodeling primes RIG-I-like receptor activation. Cell.

[170] Hage A., Bharaj P., van Tol S., Giraldo M.I., Gonzalez-Orozco M., Valerdi K.M., Warren A.N., Aguilera-Aguirre L., Xie X., Widen S.G. (2022). The RNA helicase DHX16 recognizes specific viral RNA to trigger RIG-I-dependent innate antiviral immunity. Cell Rep..

[171] Cheng J., Tao J., Li B., Shi Y., Liu H. (2023). The lncRNA HCG4 regulates the RIG-I-mediated IFN production to suppress H1N1 swine influenza virus replication. Front. Microbiol..

[172] Zhang F., Liu S., Qiao Z., Li L., Han Y., Sun J., Ge C., Zhu J., Li D., Yao H. (2024). Housekeeping U1 snRNA facilitates antiviral innate immunity by promoting TRIM25-mediated RIG-I activation. Cell Rep..

[173] Choi S.J., Lee H.C., Kim J.H., Park S.Y., Kim T.H., Lee W.K., Jang D.J., Yoon J.E., Choi Y.I., Kim S. (2016). HDAC6 regulates cellular viral RNA sensing by deacetylation of RIG-I. EMBO J..

[174] Benitez A.A., Panis M., Xue J., Varble A., Shim J.V., Frick A.L., Lopez C.B., Sachs D., tenOever B.R. (2015). In Vivo RNAi Screening Identifies MDA5 as a Significant Contributor to the Cellular Defense against Influenza A Virus. Cell Rep..

[175] Fu C., Ye S., Liu Y., Li S. (2020). Role of CARD Region of MDA5 Gene in Canine Influenza Virus Infection. Viruses.

[176] Shao Q., Fu F., Zhu P., Yu X., Wang J., Wang Z., Ma J., Wang H., Yan Y., Cheng Y. (2023). Pigeon MDA5 inhibits viral replication by triggering antiviral innate immunity. Poult. Sci..

[177] Liniger M., Summerfield A., Zimmer G., McCullough K.C., Ruggli N. (2012). Chicken cells sense influenza A virus infection through MDA5 and CARDIF signaling involving LGP2. J. Virol..

[178] Hayashi T., Watanabe C., Suzuki Y., Tanikawa T., Uchida Y., Saito T. (2014). Chicken MDA5 senses short double-stranded RNA with implications for antiviral response against avian influenza viruses in chicken. J. Innate Immun..

[179] Malur M., Gale M., Krug R.M. (2012). LGP2 downregulates interferon production during infection with seasonal human influenza A viruses that activate interferon regulatory factor 3. J. Virol..

[180] Si-Tahar M., Blanc F., Furio L., Chopy D., Balloy V., Lafon M., Chignard M., Fiette L., Langa F., Charneau P. (2014). Protective role of LGP2 in influenza virus pathogenesis. J. Infect. Dis..

[181] Wang Z., Choi M.K., Ban T., Yanai H., Negishi H., Lu Y., Tamura T., Takaoka A., Nishikura K., Taniguchi T. (2008). Regulation of innate immune responses by DAI (DLM-1/ZBP1) and other DNA-sensing molecules. Proc. Natl. Acad. Sci. USA.

[182] Upton J.W., Kaiser W.J., Mocarski E.S. (2012). DAI/ZBP1/DLM-1 complexes with RIP3 to mediate virus-induced programmed necrosis that is targeted by murine cytomegalovirus vIRA. Cell Host Microbe.

[183] Wang Y., Hao Q., Florence J.M., Jung B.G., Kurdowska A.K., Samten B., Idell S., Tang H. (2019). Influenza Virus Infection Induces ZBP1 Expression and Necroptosis in Mouse Lungs. Front. Cell. Infect. Microbiol..

[184] Wang X., Xiong J., Zhou D., Zhang S., Wang L., Tian Q., Li C., Liu J., Wu Y., Li J. (2022). TRIM34 modulates influenza virus-activated programmed cell death by targeting Z-DNA-binding protein 1 for K63-linked polyubiquitination. J. Biol. Chem..

[185] Nogusa S., Thapa R.J., Dillon C.P., Liedmann S., Oguin T.H., Ingram J.P., Rodriguez D.A., Kosoff R., Sharma S., Sturm O. (2016). RIPK3 Activates Parallel Pathways of MLKL-Driven Necroptosis and FADD-Mediated Apoptosis to Protect against Influenza A Virus. Cell Host Microbe.

[186] Zheng M., Karki R., Vogel P., Kanneganti T.D. (2020). Caspase-6 Is a Key Regulator of Innate Immunity, Inflammasome Activation, and Host Defense. Cell.

[187] Malireddi R.K.S., Sharma B.R., Bynigeri R.R., Wang Y., Lu J., Kanneganti T.D. (2023). ZBP1 Drives IAV-Induced NLRP3 Inflammasome Activation and Lytic Cell Death, PANoptosis, Independent of the Necroptosis Executioner MLKL. Viruses.

[188] Lei X., Chen Y., Lien E., Fitzgerald K.A. (2023). MLKL-Driven Inflammasome Activation and Caspase-8 Mediate Inflammatory Cell Death in Influenza A Virus Infection. mBio.

[189] Kelley N., Jeltema D., Duan Y., He Y. (2019). The NLRP3 Inflammasome: An Overview of Mechanisms of Activation and Regulation. Int. J. Mol. Sci..

[190] Wan P., Zhang S., Ruan Z., Liu X., Yang G., Jia Y., Li Y., Pan P., Wang W., Li G. (2022). AP-1 signaling pathway promotes pro-IL-1beta transcription to facilitate NLRP3 inflammasome activation upon influenza A virus infection. Virulence.

[191] Ichinohe T., Lee H.K., Ogura Y., Flavell R., Iwasaki A. (2009). Inflammasome recognition of influenza virus is essential for adaptive immune responses. J. Exp. Med..

[192] Christgen S., Place D.E., Zheng M., Briard B., Yamamoto M., Kanneganti T.D. (2022). The IFN-inducible GTPase IRGB10 regulates viral replication and inflammasome activation during influenza A virus infection in mice. Eur. J. Immunol..

[193] Pothlichet J., Meunier I., Davis B.K., Ting J.P., Skamene E., von Messling V., Vidal S.M. (2013). Type I IFN triggers RIG-I/TLR3/NLRP3-dependent inflammasome activation in influenza A virus infected cells. PLoS Pathog..

[194] Wang X., Jiang W., Yan Y., Gong T., Han J., Tian Z., Zhou R. (2014). RNA viruses promote activation of the NLRP3 inflammasome through a RIP1-RIP3-DRP1 signaling pathway. Nat. Immunol..

[195] Park H.S., Liu G., Liu Q., Zhou Y. (2018). Swine Influenza Virus Induces RIPK1/DRP1-Mediated Interleukin-1 Beta Production. Viruses.

[196] Kesavardhana S., Samir P., Zheng M., Malireddi R.K.S., Karki R., Sharma B.R., Place D.E., Briard B., Vogel P., Kanneganti T.D. (2021). DDX3X coordinates host defense against influenza virus by activating the NLRP3 inflammasome and type I interferon response. J. Biol. Chem..

[197] Park H.S., Lu Y., Pandey K., Liu G., Zhou Y. (2021). NLRP3 Inflammasome Activation Enhanced by TRIM25 is Targeted by the NS1 Protein of 2009 Pandemic Influenza A Virus. Front. Microbiol..

[198] Ichinohe T., Yamazaki T., Koshiba T., Yanagi Y. (2013). Mitochondrial protein mitofusin 2 is required for NLRP3 inflammasome activation after RNA virus infection. Proc. Natl. Acad. Sci. USA.

[199] Tamura T., Yanai H., Savitsky D., Taniguchi T. (2008). The IRF family transcription factors in immunity and oncogenesis. Annu. Rev. Immunol..

[200] Takahasi K., Suzuki N.N., Horiuchi M., Mori M., Suhara W., Okabe Y., Fukuhara Y., Terasawa H., Akira S., Fujita T. (2003). X-ray crystal structure of IRF-3 and its functional implications. Nat. Struct. Biol..

[201] Wang Z., Ji J., Peng D., Ma F., Cheng G., Qin F.X. (2016). Complex Regulation Pattern of IRF3 Activation Revealed by a Novel Dimerization Reporter System. J. Immunol..

[202] Jing T., Zhao B., Xu P., Gao X., Chi L., Han H., Sankaran B., Li P. (2020). The Structural Basis of IRF-3 Activation upon Phosphorylation. J. Immunol..

[203] Al Hamrashdi M., Brady G. (2022). Regulation of IRF3 activation in human antiviral signaling pathways. Biochem. Pharmacol..

[204] Panne D. (2008). The enhanceosome. Curr. Opin. Struct. Biol..

[205] Hatesuer B., Hoang H.T., Riese P., Trittel S., Gerhauser I., Elbahesh H., Geffers R., Wilk E., Schughart K. (2017). Deletion of Irf3 and Irf7 Genes in Mice Results in Altered Interferon Pathway Activation and Granulocyte-Dominated Inflammatory Responses to Influenza A Infection. J. Innate Immun..

[206] Chen X., Zhou L., Peng N., Yu H., Li M., Cao Z., Lin Y., Wang X., Li Q., Wang J. (2017). MicroRNA-302a suppresses influenza A virus-stimulated interferon regulatory factor-5 expression and cytokine storm induction. J. Biol. Chem..

[207] Irving A.T., Zhang Q., Kong P.S., Luko K., Rozario P., Wen M., Zhu F., Zhou P., Ng J.H.J., Sobota R.M. (2020). Interferon Regulatory Factors IRF1 and IRF7 Directly Regulate Gene Expression in Bats in Response to Viral Infection. Cell Rep..

[208] Wu W., Zhang W., Tian L., Brown B.R., Walters M.S., Metcalf J.P. (2020). IRF7 Is Required for the Second Phase Interferon Induction during Influenza Virus Infection in Human Lung Epithelia. Viruses.

[209] Tuerxun W., Wang Y., Cui C., Yang L., Wang S., Yu Y., Wang L. (2020). Expression pattern of the interferon regulatory factor family members in influenza virus induced local and systemic inflammatory responses. Clin. Immunol..

[210] Kim T.H., Zhou H. (2015). Functional Analysis of Chicken IRF7 in Response to dsRNA Analog Poly(I:C) by Integrating Overexpression and Knockdown. PLoS ONE.

[211] Crotta S., Davidson S., Mahlakoiv T., Desmet C.J., Buckwalter M.R., Albert M.L., Staeheli P., Wack A. (2013). Type I and type III interferons drive redundant amplification loops to induce a transcriptional signature in influenza-infected airway epithelia. PLoS Pathog..

[212] Yap G.L.R., Sachaphibulkij K., Foo S.L., Cui J., Fairhurst A.M., Lim L.H.K. (2020). Annexin-A1 promotes RIG-I-dependent signaling and apoptosis via regulation of the IRF3-IFNAR-STAT1-IFIT1 pathway in A549 lung epithelial cells. Cell Death Dis..

[213] Chen Y., Lei X., Jiang Z., Fitzgerald K.A. (2021). Cellular nucleic acid-binding protein is essential for type I interferon-mediated immunity to RNA virus infection. Proc. Natl. Acad. Sci. USA.

[214] Qian W., Wei X., Li Y., Guo K., Zou Z., Zhou H., Jin M. (2018). Duck interferon regulatory factor 1 acts as a positive regulator in duck innate antiviral response. Dev. Comp. Immunol..

[215] Kuriakose T., Zheng M., Neale G., Kanneganti T.D. (2018). IRF1 Is a Transcriptional Regulator of ZBP1 Promoting NLRP3 Inflammasome Activation and Cell Death during Influenza Virus Infection. J. Immunol..

[216] Natoli G., Saccani S., Bosisio D., Marazzi I. (2005). Interactions of NF-kappaB with chromatin: The art of being at the right place at the right time. Nat. Immunol..

[217] Hayden M.S., Ghosh S. (2008). Shared principles in NF-kappaB signaling. Cell.

[218] Taniguchi K., Karin M. (2018). NF-kappaB, inflammation, immunity and cancer: Coming of age. Nat. Rev. Immunol..

[219] Zhang L., Ye X., Liu Y., Zhang Z., Xia X., Dong S. (2023). Research progress on the effect of traditional Chinese medicine on the activation of PRRs-mediated NF-kappaB signaling pathway to inhibit influenza pneumonia. Front. Pharmacol..

[220] Reis A.L., McCauley J.W. (2013). The influenza virus protein PB1-F2 interacts with IKKbeta and modulates NF-kappaB signalling. PLoS ONE.

[221] Munir M., Embry A., Doench J.G., Heaton N.S., Wilen C.B., Orchard R.C. (2024). Genome-wide CRISPR activation screen identifies JADE3 as an antiviral activator of NF-kB-dependent IFITM3 expression. J. Biol. Chem..

[222] Rashid F., Xie Z., Li M., Xie Z., Luo S., Xie L. (2023). Roles and functions of IAV proteins in host immune evasion. Front. Immunol..

[223] Killip M.J., Fodor E., Randall R.E. (2015). Influenza virus activation of the interferon system. Virus Res..

[224] Husain M. (2024). Influenza Virus Host Restriction Factors: The ISGs and Non-ISGs. Pathogens.

[225] Koerner I., Kochs G., Kalinke U., Weiss S., Staeheli P. (2007). Protective role of beta interferon in host defense against influenza A virus. J. Virol..

[226] Mordstein M., Kochs G., Dumoutier L., Renauld J.C., Paludan S.R., Klucher K., Staeheli P. (2008). Interferon-lambda contributes to innate immunity of mice against influenza A virus but not against hepatotropic viruses. PLoS Pathog..

[227] Jewell N.A., Cline T., Mertz S.E., Smirnov S.V., Flano E., Schindler C., Grieves J.L., Durbin R.K., Kotenko S.V., Durbin J.E. (2010). Lambda interferon is the predominant interferon induced by influenza A virus infection in vivo. J. Virol..

[228] Galani I.E., Triantafyllia V., Eleminiadou E.E., Koltsida O., Stavropoulos A., Manioudaki M., Thanos D., Doyle S.E., Kotenko S.V., Thanopoulou K. (2017). Interferon-lambda Mediates Non-redundant Front-Line Antiviral Protection against Influenza Virus Infection without Compromising Host Fitness. Immunity.

[229] Philips R.L., Wang Y., Cheon H., Kanno Y., Gadina M., Sartorelli V., Horvath C.M., Darnell J.E., Stark G.R., O’Shea J.J. (2022). The JAK-STAT pathway at 30: Much learned, much more to do. Cell.

[230] Morris R., Kershaw N.J., Babon J.J. (2018). The molecular details of cytokine signaling via the JAK/STAT pathway. Protein Sci..

[231] Stark G.R., Darnell J.E. (2012). The JAK-STAT pathway at twenty. Immunity.

[232] Rengachari S., Groiss S., Devos J.M., Caron E., Grandvaux N., Panne D. (2018). Structural basis of STAT2 recognition by IRF9 reveals molecular insights into ISGF3 function. Proc. Natl. Acad. Sci. USA.

[233] Mahony R., Gargan S., Roberts K.L., Bourke N., Keating S.E., Bowie A.G., O’Farrelly C., Stevenson N.J. (2017). A novel anti-viral role for STAT3 in IFN-alpha signalling responses. Cell. Mol. Life Sci..

[234] Liu S., Liao Y., Chen B., Chen Y., Yu Z., Wei H., Zhang L., Huang S., Rothman P.B., Gao G.F. (2021). Critical role of Syk-dependent STAT1 activation in innate antiviral immunity. Cell Rep..

[235] Li X., Liu S., Rai K.R., Zhou W., Wang S., Chi X., Guo G., Chen J.L., Liu S. (2022). Initial activation of STAT2 induced by IAV infection is critical for innate antiviral immunity. Front. Immunol..

[236] Liu S., Liu S., Yu Z., Zhou W., Zheng M., Gu R., Hong J., Yang Z., Chi X., Guo G. (2023). STAT3 regulates antiviral immunity by suppressing excessive interferon signaling. Cell Rep..

[237] Hermesh T., Moran T.M., Jain D., Lopez C.B. (2012). Granulocyte colony-stimulating factor protects mice during respiratory virus infections. PLoS ONE.

[238] Lauder S.N., Jones E., Smart K., Bloom A., Williams A.S., Hindley J.P., Ondondo B., Taylor P.R., Clement M., Fielding C. (2013). Interleukin-6 limits influenza-induced inflammation and protects against fatal lung pathology. Eur. J. Immunol..

[239] Liu B., Mori I., Hossain M.J., Dong L., Takeda K., Kimura Y. (2004). Interleukin-18 improves the early defence system against influenza virus infection by augmenting natural killer cell-mediated cytotoxicity. J. Gen. Virol..

[240] Niu J., Wu S., Chen M., Xu K., Guo Q., Lu A., Zhao L., Sun B., Meng G. (2019). Hyperactivation of the NLRP3 inflammasome protects mice against influenza A virus infection via IL-1beta mediated neutrophil recruitment. Cytokine.

[241] Denton A.E., Doherty P.C., Turner S.J., La Gruta N.L. (2007). IL-18, but not IL-12, is required for optimal cytokine production by influenza virus-specific CD8+ T cells. Eur. J. Immunol..

[242] Schmitz N., Kurrer M., Bachmann M.F., Kopf M. (2005). Interleukin-1 is responsible for acute lung immunopathology but increases survival of respiratory influenza virus infection. J. Virol..

[243] Pang I.K., Ichinohe T., Iwasaki A. (2013). IL-1R signaling in dendritic cells replaces pattern-recognition receptors in promoting CD8(+) T cell responses to influenza A virus. Nat. Immunol..

[244] Ji S., Dai M.Y., Huang Y., Ren X.C., Jiang M.L., Qiao J.P., Zhang W.Y., Xu Y.H., Shen J.L., Zhang R.Q. (2022). Influenza a virus triggers acute exacerbation of chronic obstructive pulmonary disease by increasing proinflammatory cytokines secretion via NLRP3 inflammasome activation. J. Inflamm..

[245] Ren R., Wu S., Cai J., Yang Y., Ren X., Feng Y., Chen L., Qin B., Xu C., Yang H. (2017). The H7N9 influenza A virus infection results in lethal inflammation in the mammalian host via the NLRP3-caspase-1 inflammasome. Sci. Rep..

[246] Rosli S., Harpur C.M., Lam M., West A.C., Hodges C., Mansell A., Lawlor K.E., Tate M.D. (2023). Gasdermin D promotes hyperinflammation and immunopathology during severe influenza A virus infection. Cell Death Dis..

[247] Ji Z.X., Wang X.Q., Liu X.F. (2021). NS1: A Key Protein in the “Game” Between Influenza A Virus and Host in Innate Immunity. Front. Cell. Infect. Microbiol..

[248] Zhang L., Wang J., Munoz-Moreno R., Kim M., Sakthivel R., Mo W., Shao D., Anantharaman A., Garcia-Sastre A., Conrad N.K. (2018). Influenza Virus NS1 Protein-RNA Interactome Reveals Intron Targeting. J. Virol..

[249] Kumari R., Guo Z., Kumar A., Wiens M., Gangappa S., Katz J.M., Cox N.J., Lal R.B., Sarkar D., Fisher P.B. (2020). Influenza virus NS1-C/EBPbeta gene regulatory complex inhibits RIG-I transcription. Antivir. Res..

[250] Sun X., Feng W., Guo Y., Wang Q., Dong C., Zhang M., Guan Z., Duan M. (2018). MCPIP1 attenuates the innate immune response to influenza A virus by suppressing RIG-I expression in lung epithelial cells. J. Med. Virol..

[251] Guo Z., Chen L.M., Zeng H., Gomez J.A., Plowden J., Fujita T., Katz J.M., Donis R.O., Sambhara S. (2007). NS1 protein of influenza A virus inhibits the function of intracytoplasmic pathogen sensor, RIG-I. Am. J. Respir. Cell Mol. Biol..

[252] Mibayashi M., Martinez-Sobrido L., Loo Y.M., Cardenas W.B., Gale M., Garcia-Sastre A. (2007). Inhibition of retinoic acid-inducible gene I-mediated induction of beta interferon by the NS1 protein of influenza A virus. J. Virol..

[253] Jureka A.S., Kleinpeter A.B., Cornilescu G., Cornilescu C.C., Petit C.M. (2015). Structural Basis for a Novel Interaction between the NS1 Protein Derived from the 1918 Influenza Virus and RIG-I. Structure.

[254] Jureka A.S., Kleinpeter A.B., Tipper J.L., Harrod K.S., Petit C.M. (2020). The influenza NS1 protein modulates RIG-I activation via a strain-specific direct interaction with the second CARD of RIG-I. J. Biol. Chem..

[255] Rajsbaum R., Albrecht R.A., Wang M.K., Maharaj N.P., Versteeg G.A., Nistal-Villan E., Garcia-Sastre A., Gack M.U. (2012). Species-specific inhibition of RIG-I ubiquitination and IFN induction by the influenza A virus NS1 protein. PLoS Pathog..

[256] Gack M.U., Albrecht R.A., Urano T., Inn K.S., Huang I.C., Carnero E., Farzan M., Inoue S., Jung J.U., Garcia-Sastre A. (2009). Influenza A virus NS1 targets the ubiquitin ligase TRIM25 to evade recognition by the host viral RNA sensor RIG-I. Cell Host Microbe.

[257] Koliopoulos M.G., Lethier M., van der Veen A.G., Haubrich K., Hennig J., Kowalinski E., Stevens R.V., Martin S.R., Reis e Sousa C., Cusack S. (2018). Molecular mechanism of influenza A NS1-mediated TRIM25 recognition and inhibition. Nat. Commun..

[258] Evseev D., Miranzo-Navarro D., Fleming-Canepa X., Webster R.G., Magor K.E. (2022). Avian Influenza NS1 Proteins Inhibit Human, but Not Duck, RIG-I Ubiquitination and Interferon Signaling. J. Virol..

[259] Wang T., Wei F., Jiang Z., Song J., Li C., Liu J. (2022). Influenza virus NS1 interacts with 14-3-3epsilon to antagonize the production of RIG-I-mediated type I interferons. Virology.

[260] Qian W., Wei X., Guo K., Li Y., Lin X., Zou Z., Zhou H., Jin M. (2017). The C-Terminal Effector Domain of Non-Structural Protein 1 of Influenza A Virus Blocks IFN-beta Production by Targeting TNF Receptor-Associated Factor 3. Front. Immunol..

[261] Lin C.Y., Shih M.C., Chang H.C., Lin K.J., Chen L.F., Huang S.W., Yang M.L., Ma S.K., Shiau A.L., Wang J.R. (2021). Influenza a virus NS1 resembles a TRAF3-interacting motif to target the RNA sensing-TRAF3-type I IFN axis and impair antiviral innate immunity. J. Biomed. Sci..

[262] Chen W., Calvo P.A., Malide D., Gibbs J., Schubert U., Bacik I., Basta S., O’Neill R., Schickli J., Palese P. (2001). A novel influenza A virus mitochondrial protein that induces cell death. Nat. Med..

[263] Yoshizumi T., Ichinohe T., Sasaki O., Otera H., Kawabata S., Mihara K., Koshiba T. (2014). Influenza A virus protein PB1-F2 translocates into mitochondria via Tom40 channels and impairs innate immunity. Nat. Commun..

[264] Dudek S.E., Wixler L., Nordhoff C., Nordmann A., Anhlan D., Wixler V., Ludwig S. (2011). The influenza virus PB1-F2 protein has interferon antagonistic activity. Biol. Chem..

[265] Varga Z.T., Ramos I., Hai R., Schmolke M., Garcia-Sastre A., Fernandez-Sesma A., Palese P. (2011). The influenza virus protein PB1-F2 inhibits the induction of type I interferon at the level of the MAVS adaptor protein. PLoS Pathog..

[266] Xiao Y., Evseev D., Stevens C.A., Moghrabi A., Miranzo-Navarro D., Fleming-Canepa X., Tetrault D.G., Magor K.E. (2020). Influenza PB1-F2 Inhibits Avian MAVS Signaling. Viruses.

[267] Varga Z.T., Grant A., Manicassamy B., Palese P. (2012). Influenza virus protein PB1-F2 inhibits the induction of type I interferon by binding to MAVS and decreasing mitochondrial membrane potential. J. Virol..

[268] Wang R., Zhu Y., Ren C., Yang S., Tian S., Chen H., Jin M., Zhou H. (2021). Influenza A virus protein PB1-F2 impairs innate immunity by inducing mitophagy. Autophagy.

[269] Graef K.M., Vreede F.T., Lau Y.F., McCall A.W., Carr S.M., Subbarao K., Fodor E. (2010). The PB2 subunit of the influenza virus RNA polymerase affects virulence by interacting with the mitochondrial antiviral signaling protein and inhibiting expression of beta interferon. J. Virol..

[270] Patel D., Schultz L.W., Umland T.C. (2013). Influenza A polymerase subunit PB2 possesses overlapping binding sites for polymerase subunit PB1 and human MAVS proteins. Virus Res..

[271] Zeng Y., Xu S., Wei Y., Zhang X., Wang Q., Jia Y., Wang W., Han L., Chen Z., Wang Z. (2021). The PB1 protein of influenza A virus inhibits the innate immune response by targeting MAVS for NBR1-mediated selective autophagic degradation. PLoS Pathog..

[272] Liedmann S., Hrincius E.R., Guy C., Anhlan D., Dierkes R., Carter R., Wu G., Staeheli P., Green D.R., Wolff T. (2014). Viral suppressors of the RIG-I-mediated interferon response are pre-packaged in influenza virions. Nat. Commun..

[273] Soonthornvacharin S., Rodriguez-Frandsen A., Zhou Y., Galvez F., Huffmaster N.J., Tripathi S., Balasubramaniam V.R., Inoue A., de Castro E., Moulton H. (2017). Systems-based analysis of RIG-I-dependent signalling identifies KHSRP as an inhibitor of RIG-I receptor activation. Nat. Microbiol..

[274] Vogel O.A., Han J., Liang C.Y., Manicassamy S., Perez J.T., Manicassamy B. (2020). The p150 Isoform of ADAR1 Blocks Sustained RLR signaling and Apoptosis during Influenza Virus Infection. PLoS Pathog..

[275] Jiang J., Li Y., Sun Z., Gong L., Li X., Shi F., Yao J., Meng Y., Meng X., Zhang Q. (2022). LncNSPL facilitates influenza A viral immune escape by restricting TRIM25-mediated K63-linked RIG-I ubiquitination. iScience.

[276] Zhao L., Zhang X., Wu Z., Huang K., Sun X., Chen H., Jin M. (2019). The Downregulation of MicroRNA hsa-miR-340-5p in IAV-Infected A549 Cells Suppresses Viral Replication by Targeting RIG-I and OAS2. Mol. Ther. Nucleic Acids.

[277] Shi Q., Li G., Dou S., Tang L., Hou C., Wang Z., Gao Y., Gao Z., Hao Y., Mo R. (2023). Negative Regulation of RIG-I by Tim-3 Promotes H1N1 Infection. Immunol. Investig..

[278] Hussain M., Ahmed F., Henzeler B., Husain M. (2023). Anti-microbial host factor HDAC6 is antagonised by the influenza A virus through host caspases and viral PA. FEBS J..

[279] Huang K., Zhang Y., Gong W., Yang Y., Jiang L., Zhao L., Yang Y., Wei Y., Li C., He X. (2021). PGRMC1 Exerts Its Function of Anti-Influenza Virus in the Central Nervous System. Microbiol. Spectr..

[280] Talon J., Horvath C.M., Polley R., Basler C.F., Muster T., Palese P., Garcia-Sastre A. (2000). Activation of interferon regulatory factor 3 is inhibited by the influenza A virus NS1 protein. J. Virol..

[281] Zu S., Xue Q., He Z., Shi C., Wu W., Zhang J., Li W., Huang J., Jiao P., Liao M. (2020). Duck PIAS2 negatively regulates RIG-I mediated IFN-beta production by interacting with IRF7. Dev. Comp. Immunol..

[282] Zhang B., Liu M., Huang J., Zeng Q., Zhu Q., Xu S., Chen H. (2022). H1N1 Influenza A Virus Protein NS2 Inhibits Innate Immune Response by Targeting IRF7. Viruses.

[283] Yi C., Zhao Z., Wang S., Sun X., Zhang D., Sun X., Zhang A., Jin M. (2017). Influenza A Virus PA Antagonizes Interferon-beta by Interacting with Interferon Regulatory Factor 3. Front. Immunol..

[284] Wang X., Li M., Zheng H., Muster T., Palese P., Beg A.A., Garcia-Sastre A. (2000). Influenza A virus NS1 protein prevents activation of NF-kappaB and induction of alpha/beta interferon. J. Virol..

[285] Gao S., Song L., Li J., Zhang Z., Peng H., Jiang W., Wang Q., Kang T., Chen S., Huang W. (2012). Influenza A virus-encoded NS1 virulence factor protein inhibits innate immune response by targeting IKK. Cell Microbiol..

[286] Lee M.C., Yu C.P., Chen X.H., Liu M.T., Yang J.R., Chen A.Y., Huang C.H. (2022). Influenza A virus NS1 protein represses antiviral immune response by hijacking NF-kappaB to mediate transcription of type III IFN. Front. Cell. Infect. Microbiol..

[287] Jagger B.W., Wise H.M., Kash J.C., Walters K.A., Wills N.M., Xiao Y.L., Dunfee R.L., Schwartzman L.M., Ozinsky A., Bell G.L. (2012). An overlapping protein-coding region in influenza A virus segment 3 modulates the host response. Science.

[288] Hu J., Kong M., Cui Z., Gao Z., Ma C., Hu Z., Jiao X., Liu X. (2020). PA-X protein of H5N1 avian influenza virus inhibits NF-kappaB activity, a potential mechanism for PA-X counteracting the host innate immune responses. Vet. Microbiol..

[289] Feng M., Zhang Q., Wu W., Chen L., Gu S., Ye Y., Zhong Y., Huang Q., Liu S. (2021). Inducible Guanylate-Binding Protein 7 Facilitates Influenza A Virus Replication by Suppressing Innate Immunity via NF-kappaB and JAK-STAT Signaling Pathways. J. Virol..

[290] Stasakova J., Ferko B., Kittel C., Sereinig S., Romanova J., Katinger H., Egorov A. (2005). Influenza A mutant viruses with altered NS1 protein function provoke caspase-1 activation in primary human macrophages, resulting in fast apoptosis and release of high levels of interleukins 1beta and 18. J. Gen. Virol..

[291] Moriyama M., Chen I.Y., Kawaguchi A., Koshiba T., Nagata K., Takeyama H., Hasegawa H., Ichinohe T. (2016). The RNA- and TRIM25-Binding Domains of Influenza Virus NS1 Protein Are Essential for Suppression of NLRP3 Inflammasome-Mediated Interleukin-1beta Secretion. J. Virol..

[292] Tao P., Ning Z., Zhou P., Xiao W., Wang G., Li S., Zhang G. (2022). H3N2 canine influenza virus NS1 protein inhibits canine NLRP3 inflammasome activation. Vet. Immunol. Immunopathol..

[293] Chung W.C., Kang H.R., Yoon H., Kang S.J., Ting J.P., Song M.J. (2015). Influenza A Virus NS1 Protein Inhibits the NLRP3 Inflammasome. PLoS ONE.

[294] Park H.S., Liu G., Thulasi Raman S.N., Landreth S.L., Liu Q., Zhou Y. (2018). NS1 Protein of 2009 Pandemic Influenza A Virus Inhibits Porcine NLRP3 Inflammasome-Mediated Interleukin-1 Beta Production by Suppressing ASC Ubiquitination. J. Virol..

[295] Boal-Carvalho I., Mazel-Sanchez B., Silva F., Garnier L., Yildiz S., Bonifacio J.P., Niu C., Williams N., Francois P., Schwerk N. (2020). Influenza A viruses limit NLRP3-NEK7-complex formation and pyroptosis in human macrophages. EMBO Rep..

[296] Cheung P.H., Ye Z.W., Lee T.T., Chen H., Chan C.P., Jin D.Y. (2020). PB1-F2 protein of highly pathogenic influenza A (H7N9) virus selectively suppresses RNA-induced NLRP3 inflammasome activation through inhibition of MAVS-NLRP3 interaction. J. Leukoc. Biol..

[297] Silva F., Boal-Carvalho I., Williams N., Chabert M., Niu C., Hedhili D., Choltus H., Liaudet N., Gaia N., Karenovics W. (2024). Identification of a short sequence motif in the influenza A virus pathogenicity factor PB1-F2 required for inhibition of human NLRP3. J. Virol..

[298] Jia D., Rahbar R., Chan R.W., Lee S.M., Chan M.C., Wang B.X., Baker D.P., Sun B., Peiris J.S., Nicholls J.M. (2010). Influenza virus non-structural protein 1 (NS1) disrupts interferon signaling. PLoS ONE.

[299] Pauli E.K., Schmolke M., Wolff T., Viemann D., Roth J., Bode J.G., Ludwig S. (2008). Influenza A virus inhibits type I IFN signaling via NF-kappaB-dependent induction of SOCS-3 expression. PLoS Pathog..

[300] Du Y., Yang F., Wang Q., Xu N., Xie Y., Chen S., Qin T., Peng D. (2020). Influenza a virus antagonizes type I and type II interferon responses via SOCS1-dependent ubiquitination and degradation of JAK1. Virol. J..

[301] Yang H., Dong Y., Bian Y., Xu N., Wu Y., Yang F., Du Y., Qin T., Chen S., Peng D. (2022). The influenza virus PB2 protein evades antiviral innate immunity by inhibiting JAK1/STAT signalling. Nat. Commun..

[302] Xia C., Vijayan M., Pritzl C.J., Fuchs S.Y., McDermott A.B., Hahm B. (2015). Hemagglutinin of Influenza A Virus Antagonizes Type I Interferon (IFN) Responses by Inducing Degradation of Type I IFN Receptor 1. J. Virol..

[303] Xia C., Wolf J.J., Vijayan M., Studstill C.J., Ma W., Hahm B. (2018). Casein Kinase 1alpha Mediates the Degradation of Receptors for Type I and Type II Interferons Caused by Hemagglutinin of Influenza A Virus. J. Virol..

[304] Lin X., Yu S., Ren P., Sun X., Jin M. (2020). Human microRNA-30 inhibits influenza virus infection by suppressing the expression of SOCS1, SOCS3, and NEDD4. Cell Microbiol..

[305] Guo M., Li F., Ji J., Liu Y., Liu F., Zhao Y., Li J., Han S., Wang Q., Ding G. (2020). Inhibition of miR-93 promotes interferon effector signaling to suppress influenza A infection by upregulating JAK1. Int. Immunopharmacol..

[306] Othumpangat S., Beezhold D.H., Umbright C.M., Noti J.D. (2021). Influenza Virus-Induced Novel miRNAs Regulate the STAT Pathway. Viruses.

[307] Katze M.G., Tomita J., Black T., Krug R.M., Safer B., Hovanessian A. (1988). Influenza virus regulates protein synthesis during infection by repressing autophosphorylation and activity of the cellular 68,000-Mr protein kinase. J. Virol..

[308] Lu Y., Wambach M., Katze M.G., Krug R.M. (1995). Binding of the influenza virus NS1 protein to double-stranded RNA inhibits the activation of the protein kinase that phosphorylates the elF-2 translation initiation factor. Virology.

[309] Bergmann M., Garcia-Sastre A., Carnero E., Pehamberger H., Wolff K., Palese P., Muster T. (2000). Influenza virus NS1 protein counteracts PKR-mediated inhibition of replication. J. Virol..

[310] Min J.Y., Krug R.M. (2006). The primary function of RNA binding by the influenza A virus NS1 protein in infected cells: Inhibiting the 2′-5′ oligo (A) synthetase/RNase L pathway. Proc. Natl. Acad. Sci. USA.

[311] Zhu Z., Shi Z., Yan W., Wei J., Shao D., Deng X., Wang S., Li B., Tong G., Ma Z. (2013). Nonstructural protein 1 of influenza A virus interacts with human guanylate-binding protein 1 to antagonize antiviral activity. PLoS ONE.

[312] Hu S., Yin L., Mei S., Li J., Xu F., Sun H., Liu X., Cen S., Liang C., Li A. (2017). BST-2 restricts IAV release and is countered by the viral M2 protein. Biochem. J..

[313] Chesarino N.M., McMichael T.M., Yount J.S. (2015). E3 Ubiquitin Ligase NEDD4 Promotes Influenza Virus Infection by Decreasing Levels of the Antiviral Protein IFITM3. PLoS Pathog..

[314] Shan J., Zhao B., Shan Z., Nie J., Deng R., Xiong R., Tsun A., Pan W., Zhao H., Chen L. (2017). Histone demethylase LSD1 restricts influenza A virus infection by erasing IFITM3-K88 monomethylation. PLoS Pathog..

[315] Esposito S., Molteni C.G., Giliani S., Mazza C., Scala A., Tagliaferri L., Pelucchi C., Fossali E., Plebani A., Principi N. (2012). Toll-like receptor 3 gene polymorphisms and severity of pandemic A/H1N1/2009 influenza in otherwise healthy children. Virol. J..

[316] Lee N., Cao B., Ke C., Lu H., Hu Y., Tam C.H.T., Ma R.C.W., Guan D., Zhu Z., Li H. (2017). IFITM3, TLR3, and CD55 Gene SNPs and Cumulative Genetic Risks for Severe Outcomes in Chinese Patients With H7N9/H1N1pdm09 Influenza. J. Infect. Dis..

[317] Lim H.K., Huang S.X.L., Chen J., Kerner G., Gilliaux O., Bastard P., Dobbs K., Hernandez N., Goudin N., Hasek M.L. (2019). Severe influenza pneumonitis in children with inherited TLR3 deficiency. J. Exp. Med..

[318] Pryimenko N.O., Kotelevska T.M., Koval T.I., Syzova L.M., Dubynska H.M., Kaidashev Icapital Er C. (2019). Genetic polymorphism ARG753GLN of TLR-2, LEU412PHE of TLR-3, ASP299GLY of TLR-4 in patients with influenza and influenza-associated pneumonia. Wiad. Lek..

[319] Bucciol G., Desmet L., Corveleyn A., Meyts I., Leuven Laboratory of Inborn Errors of Immunity (2022). Pathogenic P554S Variant in TLR3 in a Patient with Severe Influenza Pneumonia. J. Clin. Immunol..

[320] Choudhary M.L., Chaudhary U., Salve M., Shinde P., Padbidri V., Sangle S.A., Salvi S., Bavdekar A.R., D’Costa P., Alagarasu K. (2022). Functional Single-Nucleotide Polymorphisms in the MBL2 and TLR3 Genes Influence Disease Severity in Influenza A (H1N1)pdm09 Virus-Infected Patients from Maharashtra, India. Viral Immunol..

[321] Jorgensen S.E., Christiansen M., Ryo L.B., Gad H.H., Gjedsted J., Staeheli P., Mikkelsen J.G., Storgaard M., Hartmann R., Mogensen T.H. (2018). Defective RNA sensing by RIG-I in severe influenza virus infection. Clin. Exp. Immunol..

[322] Lee S., Zhang Y., Newhams M., Novak T., Thomas P.G., Mourani P.M., Hall M.W., Loftis L.L., Cvijanovich N.Z., Tarquinio K.M. (2022). DDX58 Is Associated With Susceptibility to Severe Influenza Virus Infection in Children and Adolescents. J. Infect. Dis..

[323] Lee M.N., Ye C., Villani A.C., Raj T., Li W., Eisenhaure T.M., Imboywa S.H., Chipendo P.I., Ran F.A., Slowikowski K. (2014). Common genetic variants modulate pathogen-sensing responses in human dendritic cells. Science.

[324] Ciancanelli M.J., Huang S.X., Luthra P., Garner H., Itan Y., Volpi S., Lafaille F.G., Trouillet C., Schmolke M., Albrecht R.A. (2015). Infectious disease. Life-threatening influenza and impaired interferon amplification in human IRF7 deficiency. Science.

[325] Hernandez N., Melki I., Jing H., Habib T., Huang S.S.Y., Danielson J., Kula T., Drutman S., Belkaya S., Rattina V. (2018). Life-threatening influenza pneumonitis in a child with inherited IRF9 deficiency. J. Exp. Med..

[326] Thomsen M.M., Jorgensen S.E., Gad H.H., Storgaard M., Gjedsted J., Christiansen M., Hartmann R., Mogensen T.H. (2019). Defective interferon priming and impaired antiviral responses in a patient with an IRF7 variant and severe influenza. Med. Microbiol. Immunol..

[327] Calik Basaran N., Tan C., Ozisik L., Ozbek B., Inkaya A.C., Alp S., Ersoy E.O., Ayvaz D.C., Tezcan I. (2022). Evaluation of the peripheral blood T and B cell subsets and IRF-7 variants in adult patients with severe influenza virus infection. Health Sci. Rep..

[328] Chen Y., Graf L., Chen T., Liao Q., Bai T., Petric P.P., Zhu W., Yang L., Dong J., Lu J. (2021). Rare variant MX1 alleles increase human susceptibility to zoonotic H7N9 influenza virus. Science.

[329] Graf L., Dick A., Sendker F., Barth E., Marz M., Daumke O., Kochs G. (2018). Effects of allelic variations in the human myxovirus resistance protein A on its antiviral activity. J. Biol. Chem..

[330] Mitchell P.S., Patzina C., Emerman M., Haller O., Malik H.S., Kochs G. (2012). Evolution-guided identification of antiviral specificity determinants in the broadly acting interferon-induced innate immunity factor MxA. Cell Host Microbe.

[331] Dittmann J., Stertz S., Grimm D., Steel J., Garcia-Sastre A., Haller O., Kochs G. (2008). Influenza A virus strains differ in sensitivity to the antiviral action of Mx-GTPase. J. Virol..

[332] Zimmermann P., Manz B., Haller O., Schwemmle M., Kochs G. (2011). The viral nucleoprotein determines Mx sensitivity of influenza A viruses. J. Virol..

[333] Manz B., Dornfeld D., Gotz V., Zell R., Zimmermann P., Haller O., Kochs G., Schwemmle M. (2013). Pandemic influenza A viruses escape from restriction by human MxA through adaptive mutations in the nucleoprotein. PLoS Pathog..

[334] Riegger D., Hai R., Dornfeld D., Manz B., Leyva-Grado V., Sanchez-Aparicio M.T., Albrecht R.A., Palese P., Haller O., Schwemmle M. (2015). The nucleoprotein of newly emerged H7N9 influenza A virus harbors a unique motif conferring resistance to antiviral human MxA. J. Virol..

[335] Gotz V., Magar L., Dornfeld D., Giese S., Pohlmann A., Hoper D., Kong B.W., Jans D.A., Beer M., Haller O. (2016). Influenza A viruses escape from MxA restriction at the expense of efficient nuclear vRNP import. Sci. Rep..

[336] Deeg C.M., Hassan E., Mutz P., Rheinemann L., Gotz V., Magar L., Schilling M., Kallfass C., Nurnberger C., Soubies S. (2017). In vivo evasion of MxA by avian influenza viruses requires human signature in the viral nucleoprotein. J. Exp. Med..

[337] Dornfeld D., Petric P.P., Hassan E., Zell R., Schwemmle M. (2019). Eurasian Avian-Like Swine Influenza A Viruses Escape Human MxA Restriction through Distinct Mutations in Their Nucleoprotein. J. Virol..

[338] Everitt A.R., Clare S., Pertel T., John S.P., Wash R.S., Smith S.E., Chin C.R., Feeley E.M., Sims J.S., Adams D.J. (2012). IFITM3 restricts the morbidity and mortality associated with influenza. Nature.

[339] Allen E.K., Randolph A.G., Bhangale T., Dogra P., Ohlson M., Oshansky C.M., Zamora A.E., Shannon J.P., Finkelstein D., Dressen A. (2017). SNP-mediated disruption of CTCF binding at the IFITM3 promoter is associated with risk of severe influenza in humans. Nat. Med..

[340] Zhang Y.H., Zhao Y., Li N., Peng Y.C., Giannoulatou E., Jin R.H., Yan H.P., Wu H., Liu J.H., Liu N. (2013). Interferon-induced transmembrane protein-3 genetic variant rs12252-C is associated with severe influenza in Chinese individuals. Nat. Commun..

[341] Pan Y., Yang P., Dong T., Zhang Y., Shi W., Peng X., Cui S., Zhang D., Lu G., Liu Y. (2017). IFITM3 Rs12252-C Variant Increases Potential Risk for Severe Influenza Virus Infection in Chinese Population. Front. Cell. Infect. Microbiol..

[342] Kim Y.C., Jeong M.J., Jeong B.H. (2020). Strong association of regulatory single nucleotide polymorphisms (SNPs) of the IFITM3 gene with influenza H1N1 2009 pandemic virus infection. Cell Mol. Immunol..

[343] Wang Z., Zhang A., Wan Y., Liu X., Qiu C., Xi X., Ren Y., Wang J., Dong Y., Bao M. (2014). Early hypercytokinemia is associated with interferon-induced transmembrane protein-3 dysfunction and predictive of fatal H7N9 infection. Proc. Natl. Acad. Sci. USA.

[344] Xuan Y., Wang L.N., Li W., Zi H.R., Guo Y., Yan W.J., Chen X.B., Wei P.M. (2015). IFITM3 rs12252 T>C polymorphism is associated with the risk of severe influenza: A meta-analysis. Epidemiol. Infect..

[345] Kim Y.C., Jeong B.H. (2020). Ethnic variation in risk genotypes based on single nucleotide polymorphisms (SNPs) of the interferon-inducible transmembrane 3 (IFITM3) gene, a susceptibility factor for pandemic 2009 H1N1 influenza A virus. Immunogenetics.

[346] Yang X., Tan B., Zhou X., Xue J., Zhang X., Wang P., Shao C., Li Y., Li C., Xia H. (2015). Interferon-Inducible Transmembrane Protein 3 Genetic Variant rs12252 and Influenza Susceptibility and Severity: A Meta-Analysis. PLoS ONE.

[347] Mehrbod P., Eybpoosh S., Fotouhi F., Shokouhi Targhi H., Mazaheri V., Farahmand B. (2017). Association of IFITM3 rs12252 polymorphisms, BMI, diabetes, and hypercholesterolemia with mild flu in an Iranian population. Virol. J..

[348] Jia R., Pan Q., Ding S., Rong L., Liu S.L., Geng Y., Qiao W., Liang C. (2012). The N-terminal region of IFITM3 modulates its antiviral activity by regulating IFITM3 cellular localization. J. Virol..

[349] Jia R., Xu F., Qian J., Yao Y., Miao C., Zheng Y.M., Liu S.L., Guo F., Geng Y., Qiao W. (2014). Identification of an endocytic signal essential for the antiviral action of IFITM3. Cell Microbiol..

[350] John S.P., Chin C.R., Perreira J.M., Feeley E.M., Aker A.M., Savidis G., Smith S.E., Elia A.E., Everitt A.R., Vora M. (2013). The CD225 domain of IFITM3 is required for both IFITM protein association and inhibition of influenza A virus and dengue virus replication. J. Virol..

[351] Hensen L., Matrosovich T., Roth K., Klenk H.D., Matrosovich M. (2019). HA-Dependent Tropism of H5N1 and H7N9 Influenza Viruses to Human Endothelial Cells Is Determined by Reduced Stability of the HA, Which Allows the Virus To Cope with Inefficient Endosomal Acidification and Constitutively Expressed IFITM3. J. Virol..

[352] Sanchez-Gonzalez M.T., Cienfuegos-Jimenez O., Alvarez-Cuevas S., Perez-Maya A.A., Borrego-Soto G., Marino-Martinez I.A. (2021). Prevalence of the SNP rs10774671 of the OAS1 gene in Mexico as a possible predisposing factor for RNA virus disease. Int. J. Mol. Epidemiol. Genet..

[353] Dittmann M., Hoffmann H.H., Scull M.A., Gilmore R.H., Bell K.L., Ciancanelli M., Wilson S.J., Crotta S., Yu Y., Flatley B. (2015). A serpin shapes the extracellular environment to prevent influenza A virus maturation. Cell.

[354] World Health Organization, WHO Seasonal Influenza Factsheet. https://www.who.int/news-room/fact-sheets/detail/influenza-(seasonal).

[355] Caserta L.C., Frye E.A., Butt S.L., Laverack M.A., Nooruzzaman M., Covalenda L.M., Thompson A., Prarat Koscielny M., Cronk B., Johnson A. (2024). From birds to mammals: Spillover of highly pathogenic avian influenza H5N1 virus to dairy cattle led to efficient intra- and interspecies transmission. bioRxiv.

[356] Ly H. (2024). Highly pathogenic avian influenza H5N1 virus infections of dairy cattle and livestock handlers in the United States of America. Virulence.

[357] Uyeki T.M., Milton S., Abdul Hamid C., Reinoso Webb C., Presley S.M., Shetty V., Rollo S.N., Martinez D.L., Rai S., Gonzales E.R. (2024). Highly Pathogenic Avian Influenza A(H5N1) Virus Infection in a Dairy Farm Worker. N. Engl. J. Med..

[358] Swierczynska M., Mirowska-Guzel D.M., Pindelska E. (2022). Antiviral Drugs in Influenza. Int. J. Environ. Res. Public Health.

[359] Kumari R., Sharma S.D., Kumar A., Ende Z., Mishina M., Wang Y., Falls Z., Samudrala R., Pohl J., Knight P.R. (2023). Antiviral Approaches against Influenza Virus. Clin. Microbiol. Rev..

[360] Hussain M., Galvin H.D., Haw T.Y., Nutsford A.N., Husain M. (2017). Drug resistance in influenza A virus: The epidemiology and management. Infect. Drug Resist..

[361] Kumar N., Sharma S., Kumar R., Tripathi B.N., Barua S., Ly H., Rouse B.T. (2020). Host-Directed Antiviral Therapy. Clin. Microbiol. Rev..

[362] Trimarco J.D., Heaton N.S. (2022). From high-throughput to therapeutic: Host-directed interventions against influenza viruses. Curr. Opin. Virol..

[363] Girkin J.L.N., Maltby S., Bartlett N.W. (2022). Toll-like receptor-agonist-based therapies for respiratory viral diseases: Thinking outside the cell. Eur. Respir. Rev..

[364] Kayesh M.E.H., Kohara M., Tsukiyama-Kohara K. (2023). TLR agonists as vaccine adjuvants in the prevention of viral infections: An overview. Front. Microbiol..

[365] Gautam A., Boyd D.F., Nikhar S., Zhang T., Siokas I., Van de Velde L.A., Gaevert J., Meliopoulos V., Thapa B., Rodriguez D.A. (2024). Necroptosis blockade prevents lung injury in severe influenza. Nature.

